# Association of Germline Variants in Natural Killer Cells With Tumor Immune Microenvironment Subtypes, Tumor-Infiltrating Lymphocytes, Immunotherapy Response, Clinical Outcomes, and Cancer Risk

**DOI:** 10.1001/jamanetworkopen.2019.9292

**Published:** 2019-09-04

**Authors:** Xue Xu, Jianqiang Li, Jinfeng Zou, Xiaowen Feng, Chao Zhang, Ruiqing Zheng, Weixiang Duanmu, Arnab Saha-Mandal, Zhong Ming, Edwin Wang

**Affiliations:** 1 College of Computer Science and Software Engineering, Shenzhen University, Shenzhen, China; 2 Department of Biochemistry and Molecular Biology, Cumming School of Medicine, University of Calgary, Calgary, Alberta, Canada; 3 Alberta Children’s Hospital Research Institute, University of Calgary, Calgary, Alberta, Canada; 4 Arnie Charbonneau Cancer Research Institute, University of Calgary, Calgary, Alberta, Canada; 5 University of Toronto, Toronto, Ontario, Canada; 6 Department of Biomedical Informatics, School of Basic Medical Sciences, MOE Key Lab of Cardiovascular Sciences, Peking University, Beijing, China; 7 School of Mathematical Sciences, Dalian University of Technology, Dalian, China; 8 School of Computer Science and Engineering, Central South University, Changsha, China; 9 Department of Medical Genetics, Cumming School of Medicine, University of Calgary, Calgary, Alberta, Canada; 10 Department of Oncology, Cumming School of Medicine, University of Calgary, Calgary, Alberta, Canada; 11 Department of Medicine, McGill University, Montreal, Quebec, Canada

## Abstract

**Importance:**

Only a small fraction of patients with cancer receiving immune checkpoint therapy (ICT) respond, which is associated with tumor immune microenvironment (TIME) subtypes and tumor-infiltrating lymphocytes (TILs).

**Objective:**

To examine whether germline variants of natural killer (NK) cells, a key component of the immune system, are associated with TIME subtypes, the abundance of TILs, response to ICT, clinical outcomes, and cancer risk.

**Design, Setting, and Participants:**

This genetic association study explored TIME subtypes and examined the association of the germline genomic information of patients with cancer with TIME subtypes, abundance of TILs, response to ICT, prognosis, and cancer risk. Clinical information, tumor RNA sequencing, and whole-exome sequencing (WES) data of paired normal samples of patients with 13 common cancers (n = 5883) were obtained from the Cancer Genome Atlas. The WES data of individuals with no cancer (n = 4500) were obtained from the database of Genotypes and Phenotypes. Data collection and analysis took place in March 2017.

**Main Outcomes and Measures:**

Associations between the number of germline defective genes in NK cells and survival time and the abundance of TILs.****

**Results:**

Based on tumor RNA sequencing data, tumors were stratified into TIME-rich, TIME-intermediate, and TIME-poor subtypes. Tumors of TIME-rich subtype had more TILs (TIL-NK cells in TIME-rich head and neck squamous cell carcinoma [HNSC] tumors: *t* = 4.85; 95% CI of the difference, 0.01-0.03; *P* = 2.19 × 10^−6^) compared with TIME-intermediate HNSC tumors (*t* = 3.70; 95% CI of the difference, 0.01-0.03; *P* < .001), better prognosis (patients with HNSC: hazard ratio, 0.65; 95% CI, 0.41-1.02; *P* = .054) compared with TIME-intermediate and TIME-poor subtypes, and better ICT response (patients with melanoma: odds ratio [OR], 4.45; 95% CI, 0.99-27.08; *P* = .04). Patients with TIME-rich tumors had significantly fewer inherited defective genes in NK cells than patients with TIME-intermediate and TIME-poor tumors (patients with HNSC: OR, 0.49; 95% CI, 0.26-1.07; *P* = .005). Similarly, patients with cancer had significantly more inherited defective genes in NK cells than individuals with no cancer (patients with HNSC: OR, 19.09; 95% CI, 4.30-315.96; *P* = 6.21 × 10^−4^). Among 11 of 13 common cancers, the number of heritable defective genes in NK cells was significantly negatively associated with survival (patients with HNSC: hazard ratio, 1.77; 95% CI, 1.18-2.66; *P* = .005), abundance of TILs (patients with HNSC: *R* = −0.25; 95% CI, −0.65-2.17; *P* = 0.02), and response to ICT (patients with melanoma: OR, 4.45; 95% CI, 0.99-27.08; *P* = .04).

**Conclusions and Relevance:**

These results suggest that individuals who have more inherited defective genes in NK cells had a higher risk of developing cancer and that these inherited defects were associated with TIME subtypes, recruitment of TILs, ICT response, and clinical outcomes. The findings have implications for identifying individuals at risk for developing cancer of many types based on germline variants of NK cells and for improving existing ICT and chimeric antigen receptor–T cell therapy by adoptive transfer of healthy NK cells to patients with TIME-intermediate and TIME-poor tumors.

## Key Points

**Question:**

Are germline variants of natural killer (NK) cells associated with tumor immune microenvironment subtypes, cancer risk, prognosis, and immunotherapy?

**Findings:**

This genetic association study analyzed functionally mutated genes in the germline genomes of 5883 patients with 13 common cancers and 4500 individuals with no cancer, finding that the number of functionally mutated genes in NK cell germlines was negatively associated with the abundance of tumor-infiltrating lymphocytes, clinical outcomes, and immunotherapy response but positively associated with cancer risk.

**Meaning:**

Findings suggest that germline genetic variants in NK cells could help to identify individuals at risk of cancer and to improve existing immune checkpoint and chimeric antigen receptor–T cell therapies by adoptive transfer of healthy NK cells.

## Teaser

This genetic association study examines whether germline variants of natural killer cells are associated with tumor immune microenvironment subtypes, the abundance of tumor-infiltrating lymphocytes, response to immune checkpoint therapy, clinical outcomes, and cancer risk.

## Introduction

Over the past 2 decades, classification of tumors based on genomics has resulted in identifying distinct tumor molecular subtypes for cancer types, thereby providing a framework to study the molecular mechanisms of cancer. Tumor molecular subtypes hold the key to informing clinical outcomes and treatment. It has been well established that each cancer type has several distinct subtypes that are not shared with any other cancer type. Immune checkpoint therapy (ICT) has been able to successfully eliminate tumors in 10% to 40% of patients with many cancer types. However, in most patients, ICT has failed to have its intended effect.^1,2^ It has been thought that an understanding of tumor-infiltrating lymphocytes (TILs) and the tumor immune microenvironment (TIME) could provide insights into ICT resistance and might thereby improve existing immunotherapies.^3,4^ Therefore, with the advance of cancer immunotherapy, there is a strong interest in stratifying tumors into TIME subtypes. This stratification could provide better insights into the underlying molecular mechanisms for TILs and response to ICT as well as help to improve current immunotherapy. However, so far, there is no consensus about how to study TIMEs, and we lack efficient tools to stratify TIMEs.

The human immune system has both innate and adaptive systems. Innate lymphoid cells have a group 1 subset, which includes natural killer (NK) cells, as well as group 2 and 3 subsets.^5^ Natural killer cells are the first line of defense against tumor cells. They accurately regulate distinct germline-encoded inhibitory and activating cell surface receptors.^6^ Innate-like lymphocytes, including NK T cells and γδ T cells, also have innate features. Similar to NK cells, NK T cells and γδ T cells use cell-surface receptors to recognize tumors.^7,8^ It has been suggested that NK cells could modulate TILs.^9,10^ Germline mutations often lead to both NK deficiency (NKD) and Epstein-Barr virus–associated diseases.^11^ Further, EBV is a key risk factor for gastric cancer; almost 10% of gastric cancer cases are associated with EBV.^12^ Thus far, 45 NKD genes,^13^ such as *GATA2* (OMIM 137295),^11,12^ *GINS1 *(OMIM 610608),^6^ *IRF8* (OMIM 601565),^5^ *MCM4* (OMIM 602638),^7,8^ *RTEL1 *(OMIM 608833)^14^ and *FCGR3A* (OMIM 146740),^15,16,17^ have been clinically determined. These potential NKD genes contain many immunoreceptor tyrosine-based activation motif (ITAM)–signaling receptors, which activate NK cells by sensing ligands from TIMEs. We hypothesized that, if patients with fewer inherited defective genes in NK cells had tumors that expressed higher and more ligands of the NK cell–activating receptors, then a higher abundance of the TIL-NK cells and other TILs could be found in the TIME and vice versa.

To stratify TIMEs, we conducted an analysis of tumor RNA sequencing and whole-exome sequencing (WES) data of germlines in patients with 13 common cancers and of individuals with no cancer. We also examined the prevalence of inherited defective genes in NK cells in patients with cancer and individuals with no cancer to determine their association with the abundance of TILs and risk of cancer. Our aim was to explore TIME subtypes and examine the association of germline variants in NK cells with TIME subtypes, ICT response, and clinical outcomes.

## Methods

### Data Sets

This study followed the Strengthening the Reporting of Observational Studies in Epidemiology (STROBE) reporting guideline for a genetic association study. The Health Research Ethics Board of Alberta approved this study, deeming it exempt from review and informed consent because the data collection had already been approved. The WES data of buffy coats (n = 5883) and paired tumor RNA sequencing data (normalized with root-mean-square error based on The Cancer Genome Atlas [TCGA] data processing pipeline) of 13 common cancer types were collected from TCGA and FireBrowse. Cancer types with 200 or fewer patients were excluded. Bladder, breast, colon, glioma, head and neck, renal, lung adenocarcinoma, lung squamous cell carcinoma, prostrate, skin, stomach, thyroid, and endometrial cancers were included. There are a few primary samples of skin cancer, so only metastatic samples were included for analysis. Only estrogen receptor–positive breast cancer samples were included owing to the small sample sizes of other subtypes. Virus-infected tumors were excluded. The WES files of 4500 individuals with no cancer were downloaded from the database of Genotypes and Phenotypes (phs000473.v2.p2, phs000806.v1.p1, and phs001194.v2.p2). The RNA sequencing data of 49 ICT-trial melanoma tumors were collected from GSE91061 from Gene Expression Omnibus and of 47 gastric tumors from PRJEB25780 from the European Nucleotide Archive. We collected an NK-specific gene set to analyze NKD genes and sets of ITAM-signaling genes and ligands of the NK cell–activating receptors to analyze NK cell activity (eTable 1 and eTable 2 in the Supplement). Details of the gene set collections are described in eMethods 1, eMethods 2, and eMethods 3 in the Supplement.

### Data Processing and Analysis

Variant calling was performed using Varscan version 2.3.9 (Washington University in St Louis)^18^ based on the WES data analysis pipeline in TCGA (eMethods 4 in the Supplement). The thresholds for germline variants required variant allele fraction from 45% to 55% and above 90%. Functional variants were annotated using the Combined Annotation Dependent Depletion^19^ with the default parameters. Classification of tumors into TIME subtypes was conducted using the hierarchy clustering method (eMethods 5 in the Supplement). A deconvolution approach^20^ was used to extract the abundance of each TIL in a tumor based on tumor RNA sequencing data.

To explore the association of potential NKD genes with TIMEs, we compiled a comprehensive set of NK cell–unique, NK–NK T cell–specific, and NK–γδ T cell–specific genes (n = 157), called *NK-specific genes* (eMethods 1 in the Supplement), which was defined in the genome-wide gene expression analysis of mouse lymphocytes^14^ in ImmGen.^15^ To determine if potential NKD genes were associated with clinical outcomes, we ranked patients based on the number of mutated genes among potential NKD genes. We defined the top 30% and bottom 30% of patients as the high-NKD and low-NKD groups, respectively, and ran a log-rank test. To understand the association of inherited defective genes in NK cells with expression of ligands of the NK cell–activating receptors, abundance of TIL-NK cells, and other TILs in TIMEs, we collected known ligands (approximately 40 genes) of the NK cell–activating receptors among the potential NKD genes (eMethods 3 in the Supplement). For each cancer type, we identified the bottom 10% of patients by the number of defective genes and stratified them into 2 groups (ie, the high-ligand group and the low-ligand group) based on the ligands’ expression profiles.

To test whether TIME subtypes could guide immunotherapy, we used the significantly modulated genes of 2 pathways (ie, NK cell–mediated cytotoxicity and Wnt pathways) between TIME subtypes to assign 49 melanoma and 47 gastric clinical trial tumors into either TIME-rich or TIME-intermediate/TIME-poor subtypes using the k-nearest neighbor algorithm (eMethods 6 in the Supplement).

### Statistical Analysis

We applied *t* tests and false discovery rate to differential gene expression analysis between TIME subtypes. We applied χ^2^ tests and false discovery rate to differentially functional germline mutated gene analysis between TIME subtypes as well as between patients with cancer and individuals with no cancer. We conducted Fisher exact tests to compare the samples bearing NKD gene mutations between TIME subtypes across the 13 cancers. Log-rank tests were used for survival analysis. Pathway enrichment analysis was conducted using the Database for Annotative Visualization and Integrated Discovery (Laboratory of Human Retrovirology and Immunoinformatics)^21^ and the Kyoto Encyclopedia of Genes and Genomes pathways. For each cancer type, we identified a set of NKD genes. To test whether a set of NKD genes could be randomly identified, we conducted randomization tests (eMethods 7 in the Supplement). Statistical significance was set at *P* < .05, and all tests were 2-tailed. All analyses were performed using R version 3.4.1 (R Project for Statistical Computing).

## Results

### Universal TIME Subtypes Across 13 Common Cancers

**Figure 1.**
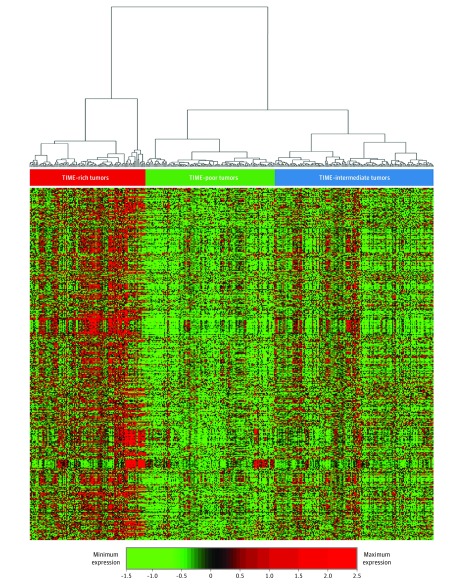
Heatmaps Showing the 3 Universal Tumor Immune Microenvironment (TIME) Subtypes A representative heatmap derived from the gene expression of immune checkpoint therapy essential genes, showing the TIME subtypes in head and neck squamous cell carcinoma. Heatmap based on log_10_ transformation of gene expression value. Green indicates minimum expression and red, maximum expression. Heatmaps for other cancer types are shown in eFigure 1 in the Supplement.

Melanoma samples were classified into 3 TIME subtypes (Figure 1; eMethods 5 in the Supplement) based on the expression of a set of genome-wide clustered regularly interspaced short palindromic repeats (CRISPR)–associated protein 9 (CRISPR–Cas9) screen-determined ICT-essential genes^22^ and other known tumor immune-related genes (n = 1294),^20,23^ which were repeatedly obtained in the 13 common cancer types (eFigure 1 in the Supplement). These results suggested that TIME subtypes were much simpler than previously defined tumor molecular subtypes. The universal TIME subtypes provided a solution for identifying the unifying features of TIME subtypes and understanding the underlying common molecular mechanisms of each TIME subtype.

**Figure 2.**
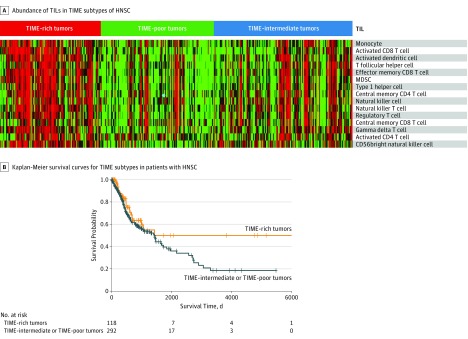
Abundance of Tumor-Infiltrating Lymphocytes (TILs) and Clinical Features of the Tumor Immune Microenvironment (TIME) Subtypes A, Heatmaps for other cancer types are shown in eFigure 2 in the Supplement. B, Kaplan-Meier survival curves of patients with the TIME-rich subtype and the combined TIME-intermediate and TIME-poor subtypes for head and neck squamous cell carcinoma (HNSC). The survival time for the patients with TIME-rich tumors was significantly longer than those with TIME-intermediate or TIME-poor subtypes. The curves for other cancer types are shown in eFigure 3 in the Supplement. CD4 indicates cluster of differentiation 4; CD56, cluster of differentiation 56; CD8, cluster of differentiation 8; and MDSC, myeloid-derived suppressor cell.

**Figure 3.**
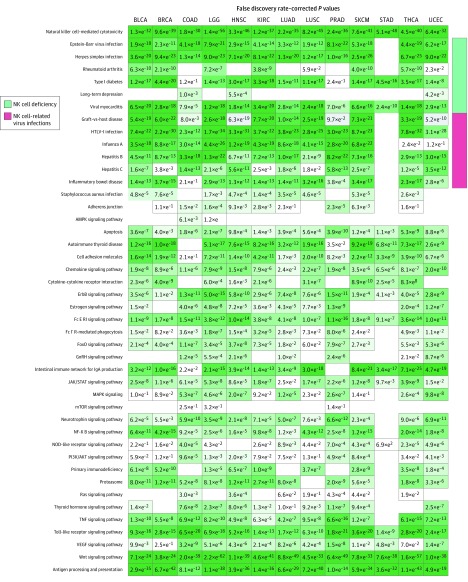
Kyoto Encyclopedia of Genes and Genomes Pathway Enrichment Analysis Heatmap shows the enriched pathways derived from the significantly differential genes of the RNA sequenced data comparing the TIME-rich subtype with the combined TIME-intermediate and TIME-poor subtypes. Columns represent Kyoto Encyclopedia of Genes and Genomes pathways, and rows represent cancer types. BLCA indicates bladder cancer; BRCA, breast cancer; COAD, colon cancer; FoxO, forkhead box protein O; GnRH, gonadotropin-releasing hormone; HNSC, head and neck squamous cell carcinoma; JAK, Janus kinases; KIRC, renal cancer; LGG, glioma cancer; LUAD, lung adenocarcinoma; LUSC, lung squamous cell cancer; NF, nuclear factor; NOD, nucleotide-binding oligomerization domain; PI3K, phosphoinositide 3-kinase; PRAD, prostate cancer; SKCM, skin cancer; STAD, stomach cancer; STAT, signal transducer and activator of transcription protein; THCA, thyroid cancer; TNF, tumor necrosis factor; UCEC, endometrial cancer; and VEGF, vascular endothelial growth factor.

To identify the characteristics of the 3 TIME subtypes, we compared their gene expression profiles, abundance of TILs, and clinical outcomes. Abundance of TILs significantly decreased from the TIME-rich subtype (TIL-NK cells in TIME-rich head and neck squamous cell carcinoma [HNSC] tumors: *t* = 4.85; 95% CI of the difference, 0.01-0.03; *P* = 2.19 × 10^−6^) to TIME-intermediate to TIME-poor subtypes (*t* = 3.70; 95% CI of the difference, 0.01-0.03; *P* < .001) (Figure 2A; eFigure 2 and eTable 3 in the Supplement). Further, patients with TIME-poor and TIME-intermediate tumors had significantly poorer prognoses than those with TIME-rich tumors (hazard ratio, 0.65; 95% CI, 0.41-1.02; *P* = .054) (Figure 2B; eFigure 3 in the Supplement), although prognoses between TIME-poor and TIME-intermediate tumors were similar. Pathway enrichment analysis of the significantly modulated genes among the TIME subtypes showed that the degree of the activated immune programs (ie, represented by the gene expression of each pathway or program), such as antigen processing and presentation (APP), NK cell–mediated cytotoxicity, and T cell pathway, was significantly decreased from TIME-rich to TIME-intermediate to TIME-poor tumors (TIME-rich HNSC tumors: *P* < .001; TIME-intermediate and TIME-poor HNSC tumors: *P* = .01) (Figure 3; eFigure 4 in the Supplement). These results were observed across the 13 common cancer types, suggesting that TIME subtypes shared common genetic and clinical features. Overall, the TIME-rich subtype represented a mean (SD) of 25.4% (8.1%) of tumors; TIME-intermediate, 32.9% (13.4%); and TIME-poor, 41.7% (15.9%) (eTable 4 in the Supplement), indicating that the TIME-rich subtype represented only a small fraction of patients. More than 70% of participants (3247 of 5373) had TIME-poor or TIME-intermediate subtypes, and most of these patients were identified as ICT nonresponders. These results could explain why only 10% to 40% of tumors (ie, depending on the type of cancer) responded to ICT.

### Associations of Inherited Defects in NK Cells With Abundance of TILs and Clinical Outcomes

The discovery of 3 universal TIME subtypes triggered us to hypothesize that TIME subtypes could be modulated by common genetic regulators. To test this hypothesis, we compared the functional germline variants (termed inherited defective genes or inherited defects) of patients across TIME subtypes, showing that inherited genetic defects were significantly decreased in patients with TIME-rich tumors compared with patients with TIME-poor and TIME-intermediate tumors (patients with TIME-rich HNSC tumors: odds ratio, 0.49; 95% CI, 0.26-1.07; *P* = .005) (eMethods 1 and eFigure 5 in the Supplement). Further pathway enrichment analysis uncovered 31 significant pathways (all false-discovery rate–corrected *P* < .05). Of those, 13 (41.9%) were associated with NK cell–deficient phenotypes, NK cell–associated virus infections, or an NK cell–mediated cytotoxicity pathway (eFigure 6 and eFigure 7 in the Supplement). For example, among the substantially differential phenotypes and pathways, 7 were known NKD phenotypes,^13,24,25^ for EBV infection, herpes simplex infection, leishmaniasis, rheumatoid arthritis, type I diabetes, long-term depression, and viral myocarditis, while 7 were NK cell–related virus infections, including graft-vs-host disease, human T-lymphotropic virus 1 infection, hepatitis B, hepatitis C, influenza A, asthma, and inflammatory bowel disease (eAppendix 2 and eFigure 16 in the Supplement). These results suggested that patients with TIME-poor and TIME-intermediate tumors have more inherited defects in NK cells than patients with TIME-rich tumors.

Based on gene expression profiles, we assigned the EBV-associated gastric tumors (in TCGA) into TIME subtypes and found that they were 6.3-fold more enriched in the TIME-intermediate and TIME-poor subtypes than in the TIME-rich subtype (odds ratio, 6.3; 95% CI, 1.1-287.6; *P* = .03). Comparing the samples bearing each NKD gene mutation between TIME subtypes across the 13 cancers, we found that most NKD genes (35 of 40 [78%]) were substantially more enriched in the TIME-intermediate and TIME-poor subtypes than in the TIME-rich subtype (eFigure 8 in the Supplement). These results suggested that known NKD genes may be associated with TIME-intermediate and TIME-poor subtypes and a lower abundance of TILs. Because they are known NKD genes, NK cell–specific gene expression for them was not considered in the above analysis.

Next, we analyzed the potential NKD genes in an NK cell–specific expressed fashion. After excluding the genes that were more highly expressed in TIME-rich tumors than TIME-intermediate and TIME-poor tumors, 13 to 22 potential NKD genes remained in each cancer type, except colon cancer (eTable 5 in the Supplement). In total, we identified 25 genes, 23 (92%) of which appeared in at least 2 cancer types. Approximately 30% (7 of 25) were NK-cell unique genes, and the rest were expressed in NK–NK T cells, NK–γδ T cells, or NK–NK T cells–γδ T cells (eFigure 9 in the Supplement).

**Figure 4.**
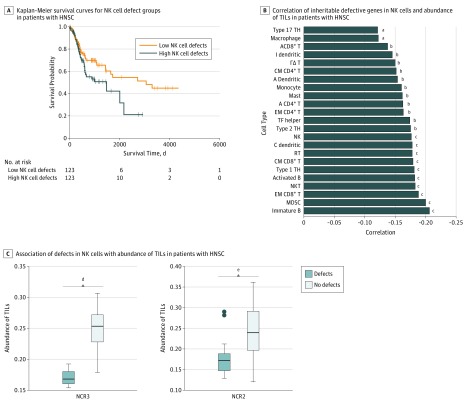
Association of Natural Killer (NK) Cells With Defective Genes With Tumor Immune Microenvironment Subtypes and Clinical Outcomes A, Representative Kaplan-Meier survival curve of the high-NKD gene patient group and low-NKD gene patient group in head and neck squamous cell carcinoma (HNSC). The curves for other cancer types are shown in eFigure 10 in the Supplement. B, Negative correlations between the number of inheritable defective genes in NK cells and the abundance of the tumor-infiltrating lymphocyte in tumor microenvironments for HNSC. The correlations for other cancer types are shown in eFigure 11 in the Supplement. C, Examples in HNSC showing that the abundance of tumor-infiltrating lymphocyte–NK cells was significantly lower in tumors bearing a defective gene in NK cells than the rest of the tumors. More examples are shown in eFigure 14 in the Supplement. Center horizontal line indicates mean; top and bottom borders of box, SD; whiskers, 95% CI; and circles, outliers. A indicates activated; B, B cell; cDC, conventional dendritic cell; CM, central memory; EM, effector memory; I, immature; MDSC, myeloid-derived suppressor cell; RT, regulatory T cell; T, T cell; TH, T helper cell; and TF helper, T follicular helper cell. ^a^*P* < .10. ^b^*P* < .05. ^c^*P* < .001. ^d^*P* = 2.12 × 10^−3^. ^e^*P* = 4.81 × 10^−3^.

In 8 (all except breast, colon, glioma, and skin) of 12 cancers (thyroid was excluded owing to a lack of survival data), the high-NKD group had significantly shorter survival than the low-NKD group (Figure 4A; eFigure 10 in the Supplement). These results suggested that more inherited defective genes in NK cells may be significantly associated with poor survival in most cancers (hazard ratio, 1.77; 95% CI, 1.18-2.66; *P* = .005). Further, in 10 of 11 cancers (except thyroid and skin) (Figure 4B; eFigure 11 in the Supplement), the abundance of TIL immune cell troops, such as NK, NK T cells, γδ T cells, CD103^+^ dendritic cells, activated CD8^+^ T cells, CD4^+^ T cells, and other immune cells, was negatively associated with the number of NK cell-defective genes (patients with HNSC: *R* = −0.21; 95% CI, −0.60-1.25; *P* = .07). These results were not random (eMethods 7 in the Supplement).

Further analyses showed that NK cell (NK T cell and/or γδ T cell)–specific receptors, such as killer-cell immunoglobulin-like receptors (ie, *KIR3DL2* and *KIR3DL3*), natural cytotoxicity receptors (ie, *NCR1*, *NCR2*, and *NCR3*), *CD244*, killer-cell lectin-like receptor F1, and killer-cell lectin-like receptor B1, represented 15 of 25 potential NKD genes (60%). Three commonly shared genes, *C17orf66*, *KHDC1*, and *KLHL30*, have an unknown function in these lineages. However, experimental studies of *CD244*, *NCR1*, and *NCR2* showed that knocking them down in NK cells affected tumor surveillance or metastasis (eTable 6 in the Supplement). Moreover, most of the potential NKD genes were NK cell–activating receptors, which depend on ITAM signaling.^17^ Thus, we hypothesized that other ITAM-signaling genes (ie, non-NK-specific ITAM genes, which had been excluded from analysis owing to their higher expression in cancer or other immune cells) in NK cells could have more inherited defective genes in TIME-poor tumors than in TIME-rich tumors and would also be associated with survival and the abundance of TILs. Indeed, the combined genes (ie, significantly defective non-NK-specific ITAM-genes and the potential NKD genes) significantly improved the association of the number of defective genes among the combined genes with 10 of 12 cancer types (ie, lower *P* values and correlation coefficients; patients with HNSC: *R* = −0.25; 95% CI, −0.65-2.17; *P* = .02) (eTable 7, eFigure 12, eFigure 13, and eAppendix 1 in the Supplement).

The strong negative correlations in Figure 4A, Figure 4B, and eFigures 10-13 in the Supplement suggest that the potential NKD genes were associated with abundance of TILs and survival in most cancers. In agreement with this conclusion, we found that the NK cells of more than 60% of patients with each cancer type had at least 1 inherited defective gene, while all patients in the top 40% had more inherited defective genes. Each potential NKD gene was associated with abundance of TILs (Figure 4C; eFigure 14 in the Supplement). The abundance of TIL-NK cells, TIL-NK T cells, TIL-γδ T cells, and TIL-CD8^+^ T cells was substantially higher in the high-ligand group than the low-ligand group of the bottom 10% of patients who had less defective genes in most cancers; however, there was no difference between the high-ligand and low-ligand groups of the top 10% of patients who had more defective genes (eTable 8 in the Supplement).

### Association of Inherited Defects in NK Cells With Tumorigenesis

**Figure 5.**
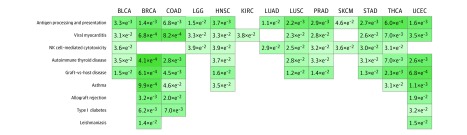
Heatmap Derived From Comparison of Individuals With No Cancer vs Patients With Cancer This heatmap shows the significantly enriched pathways derived from the significantly differential germline variants between individuals with no cancer and patients with 13 cancer types (eMethods 8 in the Supplement). Columns represent APP, NK cell pathways, and NK cell–associated phenotypes, and rows represent cancer types. BLCA indicates bladder cancer; BRCA, breast cancer; COAD, colon cancer; HNSC, head and neck squamous cell carcinoma; KIRC, renal cancer; LGG, glioma cancer; LUAD, lung adenocarcinoma; LUSC, lung squamous cell cancer; PRAD, prostate cancer; SKCM, skin cancer; STAD, stomach cancer; THCA, thyroid cancer; and UCEC, endometrial cancer.

As shown in eFigure 15 and eFigure 16 in the Supplement, significantly more inherited defective genes in NK cells were observed in patients with cancer than in individuals with no cancer. Further, we used 12 380 samples of individuals with no cancer to target-validate these results (eMethods 8 in the Supplement). We found that patients with cancer had significantly more inherited defective genes in NK cells (ie, NK cell–mediated cytotoxicity pathway and NK cell–associated phenotypes) than individuals with no cancer (HNSC: odds ratio, 19.09; 95% CI, 4.30-315.96; *P* = 6.21 × 10^−4^) (Figure 5). Furthermore, similar results were obtained when extending this analysis to 1000 randomly selected individuals with no cancer from the 1000 Genome Project.

### Association of TIME Subtypes With ICT Response

As shown in eTable 9 in the Supplement, 7 of 10 (70%) and 7 of 18 (38%) of ICT-responding melanoma and gastric tumors were assigned to the TIME-rich group, indicating that patients with TIME-rich tumors were enriched with ICT responders. Consistently, genes of the NK cell–mediated cytotoxicity pathway (melanoma tumors: false-discovery rate–corrected *P* = 2.5 × 10^−3^; gastric tumors: false-discovery rate–corrected *P* = 2.7 × 10^−3^) were expressed at a substantially lower rate in nonresponders with TIME-intermediate and TIME-poor tumors than responders with TIME-intermediate and TIME-poor tumors.

## Discussion

### A Unified View of the Tumor Immune Microenvironment

Unlike previous observations about complex tumor molecular subtypes, each of which often had its own unique features, the 3 universal TIME subtypes we identified were shared by multiple cancers, providing a framework to gain insights into the unifying features of each subtype and to understand why some tumors respond to immunotherapy. Patients with TIME-rich tumors had significantly longer survival time than patients with tumors of other TIME subtypes. The abundance of TILs gradually decreased from TIME-rich to TIME-intermediate to TIME-poor tumors. Further, APP, NK cell, and T cell signaling were more highly activated in TIME-rich than in TIME-intermediate or TIME-poor tumors. These findings suggest that TIME-poor and TIME-intermediate tumors could have a better chance of escaping immunosurveillance. Antigen processing and presentation is an immunological process for presenting antigens to T cells, and NK cell and T cell receptor pathways function as cancer cell killing. Patients who responded to ICT were more enriched with TIME-rich tumors, suggesting that adoptive NK cell transfer in combination with chimeric antigen receptor–T cell therapy or ICT could improve existing immunotherapies for nonresponders with TIME-intermediate or TIME-poor tumors. However, these conclusions were based on a small number of samples (n = 96). More samples are needed to further validate these discoveries in the future.

### Association of Potential NKD Genes With Cancer Risk, TILs, and Immunotherapy Response

The hypothesis regarding cancer immunosurveillance suggests that tumor cell transformation occurs frequently but under constant control by the immune system. Natural killer cells have a large repertoire of germline-encoded inhibitory and activating receptors to sense danger in cell surfaces. The potential NKD genes could impair NK cell function. Experimental analysis of several potential NKD genes supported this notion. Surveillance of cancerous cells and the regulation of the active immune system by NK cells have been investigated.^26,27^ A 2017 comprehensive review summarized a number of studies about NK defects and cancer development and relapse.^28^ Our findings suggest that NKD genes were associated with the recruitment of immune cells into TIMEs, and inherited defective genes in NK, NK T cells, and/or γδ T cells probably impaired communication between NK cells and other immune cells to block the recruitment of TILs. This implication was partially supported by 2 studies from 2018,^9,16^ which did not discuss inherited defects in NK cells but showed that depletion of NK cells resulted in failed recruitment of CD8^+^ T cells to TIMEs in melanoma mice. They further showed that NK cells recruited CD103^+^ dendritic cells, which in turn were required for the recruitment of CD8^+^ T cells.^9,16^ Our results suggested negative correlations between the number of NKD genes and the recruitment of CD103^+^ dendritic cells, CD8^+^ T cells, and other immune cells into TIMEs. These results suggest that individuals who bear potential NKD genes were probably at risk of developing cancer. This hypothesis is indirectly supported by a previous study^29^ showing that individuals with lower NK cytotoxic activity in peripheral blood had higher cancer incidences (n = 3500, 11-year follow-up). Similarly, previous studies showed^30,31,32^ that impaired NK cell activity was found in family members of patients with several cancers, further suggesting that the potential NKD genes are risk factors associated with cancer, abundance of TILs, TIME subtypes, and clinical outcomes. These insights provide an opportunity to identify a subpopulation who are at risk of developing cancer based on potential NKD genes. Further, adoptive NK cell transfer from healthy donors to individuals with high risk or genome correction of these potential NKD genes in the hematopoietic stem cells could postpone or prevent cancer development. This hypothesis is partially supported by a study^33^ showing that NK cell depletion in melanoma mice resulted in substantial metastasis, but adoptive transfer of NK cells protects NK cell–deficient mice from tumor establishment. In addition, our analysis suggested that type I diabetes and long-term depression phenotypes were associated with some cancers, which is also supported by nongenetic studies (eAppendix 2 and eFigure 16 in the Supplement). Alongside NK cells, we also found an association of inherited defective genes of the APP and Wnt pathways with tumorigenesis and metastasis (eFigure 17, eFigure 18, and eAppendix 3 in the Supplement).

Further, we found that approximately 30% of the substantially differential pathways and phenotypes between patients with TIME-rich tumors and those with TIME-intermediate or TIME-poor tumors were either NKD phenotypes or NK cell–related viral infection phenotypes. This result highlighted that NKD genes could be closely associated with TIME subtypes, the abundance of TILs, and survival. Furthermore, among the pathways of NK cells, APP, and Wnt, only the potential NKD genes were correlated with both survival and abundance of TILs. In 10 common cancers, potential NKD genes alone were sufficient to establish these correlations. Most patients with cancer (>60%) bear at least 1 potential NKD gene, while more than 40% of patients with cancer bear at least 3 potential NKD genes. This is consistent with the fact that more than 70% of patients with cancer do not respond to existing ICTs.

In addition, our results suggested that inherited defective genes in NK cells and the APP pathway were closely correlated with tumorigenesis. Therefore, patients with cancer could be highly selected, providing an explanation for the fact that only 11% to 21% of heavy smokers develop lung cancer in their lifetimes.^34,35^ Thus, our results suggest that germline genomes may help to determine whether a person gets cancer, and cancer is the result of interactions between high-risk germlines and risk factors. Many open questions remain (eAppendix 4 in the Supplement).

### Limitations

This study has several limitations. First, we could not perform further subgroup analyses and examine the associations between the number of NKD genes, survival time, and abundance of TILs in all cancer subtypes owing to a limited number of patient samples. More samples for each cancer type are needed to be whole-exome and RNA sequenced. Second, sample numbers for examining the association of TIME subtypes with ICT response were small. Additional trials with RNA-sequenced tumors are needed to validate the association. Third, the ICT trials used in this study administrated a single anti–programmed cell death receptor 1 agent. It is desirable to examine the association of TIME subtypes with ICT response using the trials that have used dual ICT agents.

## Conclusions

These results suggest that individuals who have more inherited defective genes in NK cells had a higher risk of developing cancer and that these inherited defects were associated with TIME subtypes, recruitment of TILs, ICT response, and clinical outcomes. Our findings open a new window to explore NK cell biology and lead to novel thinking about identifying high-risk patients for early cancer detection and immunotherapy. Strategies to harness NK cells for cancer therapy are relatively new, rapidly developing, and not used for cancer prevention yet. Insights here lead to new directions. First, they could help to identify a subpopulation at risk of developing cancer so that early cancer detection or prevention could be implemented. Second, they could help to prevent or postpone cancer development for individuals with high risk through adoptive NK cell transfer. Third, converting TIME-poor and TIME-intermediate tumors into TIME-rich tumors by manipulating NK cells or adoptive NK cell transfer could improve current ICT or chimeric antigen receptor–T cell therapy.

## Notes

Supplement. **eMethods 1. **Collecting NK Cell-Specific (NK-Specific) Genes **eMethods 2. **Immunoreceptor Tyrosine-Based Activation Motif (ITAM)–Signaling Genes **eMethods 3. **Ligands of the NK Cell Activating Receptors **eMethods 4. **Variant Calling and Functional Germline Variants **eMethods 5. **Immune Gene Set and Clustering Analysis for Determining TIME Subtypes **eMethods 6. **Assigning Immune Checkpoint Therapy (ICT) Trial Samples into TIME Subtypes **eMethods 7. **Randomization Tests of the NK-Defective Genes **eMethods 8. **Validating the Observation that Inherited Defective Genes in NK Cells and APP Pathway Were More in Patients With Cancer Than Individuals With No Cancer **eAppendix 1. **Associations of Defective Genes in NK Cell-ITAM-Signaling Genes With Clinical Outcomes and Abundance of TILs **eAppendix 2. **Inherited Defective Genes in NK Cells, Type I Diabetes, Long-term Depression Phenotypes, and Cancer **eAppendix 3. **Associations of the Inherited Defective Genes in APP and Wnt Pathways With Tumorigenesis and Metastasis **eAppendix 4. **Open Questions Remained for NK Cell Inherited Defective Genes in Cancer **eFigure 1. **Heatmaps Showing the 3 Universal TIME Subtypes **eFigure 2. **Abundance of the Tumor-Infiltrating Lymphocytes in TIME Subtypes in Cancers **eFigure 3. **Kaplan-Meier Curves Between Patients With Cancer in TIME-Rich Subtype and TIME-Intermediate and TIME-Poor Subtypes **eFigure 4. **Significantly Enriched Pathways by Comparing RNA-Seq Data in TIME-Intermediate and TIME-Poor Subtypes **eFigure 5. **Heatmaps of the Significantly Differential Functional Germline Variants Between TIME-Rich and TIME-Intermediate/TIME-Poor Subtypes **eFigure 6. **Significantly Enriched Pathways by Comparing Functional Germline Variant Between TIME-Rich and TIME-Intermediate/TIME-Poor Subtypes **eFigure 7. **Significantly Enriched Pathways of the Significantly Differential Germline Variants Between TIME-Intermediate and TIME-Poor Subtypes **eFigure 8. **A Heatmap Showing the Significantly More Inherited NKD Genes in TIME-Intermediate/TIME-Poor Subtypes Than TIME-Rich Subtype in Cancers **eFigure 9. **A Bar Chart Showing Ratios of Gene Categories of the Potential NKD Genes Across 12 Cancer Types **eFigure 10. **Kaplan-Meier Curves of the High- and Low-Number of Functionally Inherited NK Cell Variants **eFigure 11. **Negative Correlations Between the Number of the Inheritable Defective Genes and Abundance of TILs **eFigure 12. **Kaplan-Meier Curves of the High- and Low-Number of Functionally Inherited Variants of the Combined Genes **eFigure 13. **Negative Correlations Between the Number of the Inheritable Defective Combined Genes and Abundance of TILs **eFigure 14. **The Abundance of TIL-NK Cells in the Tumors Bearing a Defective Gene in NK Cells Was Significantly Lower Than the Rest of the Tumors **eFigure 15. **Heatmaps of the Significantly Differentially Functional Germline Variants Between Cancer and Cancer-Free Cohorts **eFigure 16. **Significantly Enriched Pathways Derived From the Significantly Differential Germline Variants Between Individuals With No Cancer Patients With Cancer **eFigure 17. **Kaplan-Meier Curves of the High- and Low-Number of Functionally Inherited Variants in the Wnt Signaling Pathway for Disease-Free Survival **eFigure 18. **Correlations of the Functionally Inherited Variants in the Wnt Signaling Pathway With the Abundance of TILs **eTable 1. **List of ITAM-Signaling Genes **eTable 2. **List of Ligands of the NK Activating Receptors **eTable 3. **Average Immune Cell Fractions for TIME-Rich, TIME-Intermediate, and TIME-Poor Subtypes, Respectively **eTable 4. **Fractions of the Patients in TIME-Rich, TIME-Intermediate, and TIME-Poor Subtype in Cancers **eTable 5. **NK Defective Genes in Each Cancer Type **eTable 6. **Experimental Evidence of the NK Cell Defective Genes for Tumor Surveillance **eTable 7. **ITAM-Signaling Genes Associated With Patients’ Survival and Abundance of TILs in Cancers **eTable 8. **Abundance of TILs in Tumors Stratified by the Expression of NK Cell Ligand Genes of Tumors for the Bottom 10% and Top 10% of Patients Ranked by the Number of NK-Defective Genes **eTable 9. **Clustering Analysis for the Melanoma (SKCM) and Gastric Cancer (STAD) Samples in Immune-Checkpoint Therapy (ICT) Trials **eReferences. **
