# Error in Methods

**DOI:** 10.1001/jamanetworkopen.2020.6708

**Published:** 2020-04-24

In the Original Investigation titled “Association of Germline Variants in Natural Killer Cells With Tumor Immune Microenvironment Subtypes, Tumor-Infiltrating Lymphocytes, Immunotherapy Response, Clinical Outcomes, and Cancer Risk,”^1^ published September 4, 2019, there was an error in the data sets listed in the Methods section; phs000806.v1.p1 and phs001194.v2.p2 were not used. This article has been corrected.^1^
