# Supplementary material for: Association of Germline Variants in Natural Killer Cells With Tumor Immune Microenvironment Subtypes, Tumor-Infiltrating Lymphocytes, Immunotherapy Response, Clinical Outcomes, and Cancer Risk
Source: JAMA Netw Open. 2019 Sep 4;2(9):e199292. doi: 10.1001/jamanetworkopen.2019.9292 (PMC6727785; doi:10.1001/jamanetworkopen.2019.9292)
Supplement: Supplement. — eMethods 1. Collecting NK Cell-Specific (NK-Specific) Genes eMethods 2. Immunoreceptor Tyrosine-Based Activation Motif (ITAM)–Signaling Genes eMethods 3. Ligands of the NK Cell Activating Receptors eMethods 4. Variant Calling and Functional Germline Variants eMethods 5. Immune Gene Set and Clustering Analysis for Determining TIME Subtypes eMethods 6. Assigning Immune Checkpoint Therapy (ICT) Trial Samples into TIME Subtypes eMethods 7. Randomization Tests of the NK-Defective Genes eMethods 8. Validating the Observation that Inherited Defective Genes in NK Cells and APP Pathway Were More in Patients With Cancer Than Individuals With No Cancer eAppendix 1. Associations of Defective Genes in NK Cell-ITAM-Signaling Genes With Clinical Outcomes and Abundance of TILs eAppendix 2. Inherited Defective Genes in NK Cells, Type I Diabetes, Long-term Depression Phenotypes, and Cancer eAppendix 3. Associations of the Inherited Defective Genes in APP and Wnt Pathways With Tumorigenesis and Metastasis eAppendix 4. Open Questions Remained for NK Cell Inherited Defective Genes in Cancer eFigure 1. Heatmaps Showing the 3 Universal TIME Subtypes eFigure 2. Abundance of the Tumor-Infiltrating Lymphocytes in TIME Subtypes in Cancers eFigure 3. Kaplan-Meier Curves Between Patients With Cancer in TIME-Rich Subtype and TIME-Intermediate and TIME-Poor Subtypes eFigure 4. Significantly Enriched Pathways by Comparing RNA-Seq Data in TIME-Intermediate and TIME-Poor Subtypes eFigure 5. Heatmaps of the Significantly Differential Functional Germline Variants Between TIME-Rich and TIME-Intermediate/TIME-Poor Subtypes eFigure 6. Significantly Enriched Pathways by Comparing Functional Germline Variant Between TIME-Rich and TIME-Intermediate/TIME-Poor Subtypes eFigure 7. Significantly Enriched Pathways of the Significantly Differential Germline Variants Between TIME-Intermediate and TIME-Poor Subtypes eFigure 8. A Heatmap Showing the Significantly More Inherited NKD Genes in TIME-Intermediate/TIME-Po [file jamanetwopen-2-e199292-s001.pdf]

Supplementary Online Content

Xu X, Li J, Zou J, et al. Association of germline variants in natural killer cells with tumor immune microenvironment subtypes, tumor-infiltrating lymphocytes, immunotherapy response, clinical outcomes, and cancer risk. *JAMA Netw Open*. 2019;2(9):e199292. doi:10.1001/jamanetworkopen.2019.9292

eMethods 1. Collecting NK Cell-Specific (NK-Specific) Genes
eMethods 2. Immunoreceptor Tyrosine-Based Activation Motif (ITAM)–Signaling Genes
eMethods 3. Ligands of the NK Cell Activating Receptors
eMethods 4. Variant Calling and Functional Germline Variants
eMethods 5. Immune Gene Set and Clustering Analysis for Determining TIME Subtypes
eMethods 6. Assigning Immune Checkpoint Therapy (ICT) Trial Samples into TIME Subtypes
eMethods 7. Randomization Tests of the NK-Defective Genes
eMethods 8. Validating the Observation that Inherited Defective Genes in NK Cells and APP Pathway Were More in Patients With Cancer Than Individuals With No Cancer
eAppendix 1. Associations of Defective Genes in NK Cell-ITAM-Signaling Genes With Clinical Outcomes and Abundance of TILs
eAppendix 2. Inherited Defective Genes in NK Cells, Type I Diabetes, Long-term Depression Phenotypes, and Cancer
eAppendix 3. Associations of the Inherited Defective Genes in APP and Wnt Pathways With Tumorigenesis and Metastasis
eAppendix 4. Open Questions Remained for NK Cell Inherited Defective Genes in Cancer
eFigure 1. Heatmaps Showing the 3 Universal TIME Subtypes
eFigure 2. Abundance of the Tumor-Infiltrating Lymphocytes in TIME Subtypes in Cancers
eFigure 3. Kaplan-Meier Curves Between Patients With Cancer in TIME-Rich Subtype and TIME-Intermediate and TIME-Poor Subtypes
eFigure 4. Significantly Enriched Pathways by Comparing RNA-Seq Data in TIME-Intermediate and TIME-Poor Subtypes
eFigure 5. Heatmaps of the Significantly Differential Functional Germline Variants Between TIME-Rich and TIME-Intermediate/TIME-Poor Subtypes
eFigure 6. Significantly Enriched Pathways by Comparing Functional Germline Variant Between TIME-Rich and TIME-Intermediate/TIME-Poor Subtypes
eFigure 7. Significantly Enriched Pathways of the Significantly Differential Germline Variants Between TIME-Intermediate and TIME-Poor Subtypes
eFigure 8. A Heatmap Showing the Significantly More Inherited NKD Genes in TIME-Intermediate/TIME-Poor Subtypes Than TIME-Rich Subtype in Cancers
eFigure 9. A Bar Chart Showing Ratios of Gene Categories of the Potential NKD Genes Across 12 Cancer Types

© 2019 Xu X et al. *JAMA Network Open.*

eFigure 10. Kaplan-Meier Curves of the High- and Low-Number of Functionally Inherited NK Cell Variants
eFigure 11. Negative Correlations Between the Number of the Inheritable Defective Genes and Abundance of TILs
eFigure 12. Kaplan-Meier Curves of the High- and Low-Number of Functionally Inherited Variants of the Combined Genes
eFigure 13. Negative Correlations Between the Number of the Inheritable Defective Combined Genes and Abundance of TILs
eFigure 14. The Abundance of TIL-NK Cells in the Tumors Bearing a Defective Gene in NK Cells Was Significantly Lower Than the Rest of the Tumors
eFigure 15. Heatmaps of the Significantly Differentially Functional Germline Variants Between Cancer and Cancer-Free Cohorts
eFigure 16. Significantly Enriched Pathways Derived From the Significantly Differential Germline Variants Between Individuals With No Cancer Patients With Cancer
eFigure 17. Kaplan-Meier Curves of the High- and Low-Number of Functionally Inherited Variants in the Wnt Signaling Pathway for Disease-Free Survival
eFigure 18. Correlations of the Functionally Inherited Variants in the Wnt Signaling Pathway With the Abundance of TILs
eTable 1. List of ITAM-Signaling Genes
eTable 2. List of Ligands of the NK Activating Receptors
eTable 3. Average Immune Cell Fractions for TIME-Rich, TIME-Intermediate, and TIME-Poor Subtypes, Respectively
eTable 4. Fractions of the Patients in TIME-Rich, TIME-Intermediate, and TIME-Poor Subtype in Cancers
eTable 5. NK Defective Genes in Each Cancer Type
eTable 6. Experimental Evidence of the NK Cell Defective Genes for Tumor Surveillance
eTable 7. ITAM-Signaling Genes Associated With Patients' Survival and Abundance of TILs in Cancers
eTable 8. Abundance of TILs in Tumors Stratified by the Expression of NK Cell Ligand Genes of Tumors for the Bottom 10% and Top 10% of Patients Ranked by the Number of NK-Defective Genes
eTable 9. Clustering Analysis for the Melanoma (SKCM) and Gastric Cancer (STAD) Samples in Immune-Checkpoint Therapy (ICT) Trials
eReferences.

This supplementary material has been provided by the authors to give readers additional information about their work.

eMethods 1. Collecting NK cell-specific (NK-specific) Genes

To select NK-specific genes, we used the gene signatures derived from a genome-wide gene expression analysis of mouse lymphocytes between immune cells[1] and an immune gene regulatory network (ImmGen)[2] containing the gene expression profiles of more than 300 mouse immune cell types. NK cells were reported to be easily distinguished from B cells and adaptive T cells such as CD4+ T, CD8+ T and other innate immune cells but shared many up-regulated genes with NKT and γδ T cells.[1] The shared repertoire of surface receptors, signaling molecules and transcription factors expressed by NK cells and the innate-like T cells (i.e., NKT and γδ T cells) blurs the distinctions among these cell types. Thus, we collected ~200 NK-specific genes representing (1) NK-unique genes which were more highly up-reregulated in NK cells than innate-like T cells (i.e., NKT and γδ T cells) and other immune cells (i.e., B, CD4+ T, CD8+ T and others); (2) genes (termed as NK-NKT genes here) which were highly up-regulated in both NK and NKT cells, but not in other immune cells; (3) genes (i.e., termed as NK-γδ T genes here) which were highly up-regulated in both NK and γδ T cells, but not in other immune cells; and (4) genes (i.e., termed as NK-NKT-γδ T genes here) which were highly up-regulated in NK, NKT and γδ T cells, but not in other immune cells. These genes were able to distinguish innate populations (NK, NKT and γδ T cells) from adaptive T cells, B cells and other immune cells.

We manually checked these genes from literature and gene databases such as GeneCards (https://www.genecards.org) to remove the genes which were universally expressed. Furthermore, we conducted correlation analyses between the expression of each gene and the abundance of TIL-NK, TIL-NK $CD56^{bright}$ or TIL-NK $CD56^{dim}$ cells in each cancer type. For each cancer type, the genes were remained only if they had significantly positive correlations with the abundance of any of the three TIL-NK cell subsets (FDR-corrected $p<0.05$). Finally, we obtained 157 genes (i.e., 28 of them were NK-unique genes). These genes were highly expressed on either NK cell alone, NK-NKT, NK-γδ T or NK-NKT-γδ T cells. To exclude the genes that have a

© 2019 Xu X et al. *JAMA Network Open.*

functional effect on tumor cells, we further screened the genes (i.e., from the 157 gene) which were within the bottom 30% of the expression value-ranked whole-genome genes in the TIME-rich tumors.

eMethods 2. Immunoreceptor Tyrosine-Based Activation Motif (ITAM)-Signaling Genes

Besides the NK cell specific ITAM-signaling receptors, we manually collected 18 ITAM-signaling genes (eTable 8) from literature which were expressed in NK cells and other immune cells such as T cells.

eMethods 3. Ligands of the NK Cell Activating Receptors

We manually collected 39 ligands from literature which were known as NK cell activating receptors (eTable 9).

eMethods 4. Variant Calling and Functional Germline Variants

For WES files, variant calling was performed using Varscan (version-2.3.9)[3]. Functional variants were examined and annotated using the Combined Annotation Dependent Depletion (CADD)[4] with the default parameters. To consistent with the WES data analysis pipeline in TCGA, BWA (version-0.7.15)[5] was used to align with default parameters for the cancer-free individuals, and pipe into Samtools (version-0.1.8)[6] to sort. Additional adding read groups and duplicate removal were processed with Picard-tools (version-2.6.0). The resulted BAM files were processed with GATK (version-4.0.11.0) for realignment and base recalibration.

© 2019 Xu X et al. *JAMA Network Open.*

eMethods 5. Immune Gene Set and Clustering Analysis for Determining TIME Subtypes

Immune-related genes (n=1,384) including MHC system-related genes,[7] immunophenoscore-related genes,[8] ICT essential genes for immunotherapy[9] and cytotoxic T cell-resistant genes[10] were collected and identified as critical immune-related genes (the gene pool G). RNA-sequencing data of melanoma samples were used to conduct the following analysis:

Step#1 Initialize the candidate set of key genes, that is, $G_{candidate} = \phi$

Step#2 Randomly select 30% genes from the gene set G_{random} .

Step#3 Replace the features of elements in the patient set P with G_{random} to form the sample set S_{random} .

Step#4 Group the samples S_{random} by using the hierarchical clustering method. For each of the clustering, clValid[11] was used to evaluate the clustering stability and the most stable clustering number was recorded.

Step#5 Repeat Steps 2-4 100,000 times. Rank the most stable clustering numbers and select the most suitable clustering number 3.

Step#6 Extract the genes when clustering number is 3 and rank the genes.

Select the most informative genes and record them as the final set of key-gene candidates (1,294 genes).

We then used the 1,294 genes to conduct unsupervised clustering analyses of the RNA-seq data for each cancer type to define TIME subtypes.

© 2019 Xu X et al. *JAMA Network Open.*

eMethods 6. Assigning Immune Checkpoint Therapy (ICT) Trial Samples into TIME Subtypes

ICT-clinical trial SKCM and STAD samples were assigned to the TIME subtypes derived from TCGA-SKCM and TCGA-STAD samples, respectively. To assign an ICT-clinical trial sample into a TIME subtype, t-test statistics were first conducted between TIME-rich and TIME-intermediate tumors, and between TIME-intermediate and TIME-poor tumors based on the genes derived from the NK cell-mediated cytotoxicity pathway and the Wnt signaling pathway using RNA-seq data. Spearman's correlation was then conducted between each ICT-clinical trial sample and the TCGA samples in each TIME subtype based on the obtained significantly differential genes ($p<0.05$). Finally, k-nearest neighbor algorithm (KNN, $k=5$) was used to determine the subtype of each ICT-clinical trial sample.

eMethods 7. Randomization Tests of the NK-Defective Genes

For each cancer type, we identified a set of NK-defective genes. To test if a set of NK-defective genes could be randomly identified, we conducted randomization tests. We first randomly selected 157 genes within the bottom 30% of the expression value-ranked whole-genome genes of the TIME-rich tumors for a given cancer type ($n=5,000$ times) and then employed hypergeometric tests. When a negative correlation ($p<0.05$) between the gene number in the random genes and the abundance of TIL-NK cells appeared, the hypergeometric test was marked as one success. The number of successes was calculated within the 10,000 hypergeometric tests.

eMethods 8. Validating the Observation that Inherited Defective Genes in NK Cells and APP Pathway Were More in Patients With Cancer Than Individuals With No Cancer

To further validate the observation that more inherited defective genes in NK cells were in cancer patients than cancer-free individuals, we obtained the WES data of germlines for 12,380 cancer-free samples from dbGaP (phs000473) and determined their functional mutated genes. For each cancer type, we conducted hypergeometric

© 2019 Xu X et al. *JAMA Network Open.*

tests for the NK cell-mediated cytotoxicity pathway, NK cell-associated phenotypes and the APP pathway shown in Figure 3 and eFigure 4 using the methods in our previous study.[12] Briefly, for a given cancer type, we compared cancer hallmark genes (~12,000 genes) using randomization tests to identify differentially functional germline mutated genes ($p<0.05$) between cancer and the cancer-free samples, and then conducted enrichment analyses of the NK cell-mediated cytotoxicity pathway, NK cell-associated phenotypes and APP pathway via conducting hypergeometric tests (differentially functional germline mutated genes vs the cancer hallmark genes).

eAppendix 1. Associations of Defective Genes in NK Cell-ITAM-Signaling Genes With Clinical Outcomes and Abudnance of TILs

To test whether ITAM signaling-genes in NK cells could have more inherited defects in TIME-poor tumors compared with TIME-rich tumors, we collected a set of known ITAM-dependent genes (~20 genes) in NK cells and found that some of the genes had significantly higher genetic defects in TIME-poor tumors than TIME-rich tumors (eTable 5). We further asked whether the combination of these significantly defective genes with the potentially NK cell-defective genes identified in each cancer type could improve the correlations (i.e., the negative correlations between the number of NK cell-defective genes and survival and TILs' abundance). We showed that the correlations were significantly improved (i.e., lower p values and correlation coefficients) in 11 out of the 12 cancer types except COAD. These results suggested that inheritably genetic defects in ITAM-signalling genes of the NK cells could highly associated with TILs' abundance and survival in all the cancers except COAD in a synergy manner. NK cells surrounding COAD tumors enabled to directly interact with environmental factors such as microbiome, drinks, and others so that COAD associated NK cells were much more complex than other cancers.

eAppendix 2. Inherited Defective Genes in NK cells, Type I Diabetes, Long-term Depression Phenotypes, and Cancer

© 2019 Xu X et al. *JAMA Network Open.*

Germline genomic analysis here suggested that Type I diabetes and long-term depression phenotypes were linked to some cancers. This is supported by non-genetic studies showing that diabetes is a risk factor for all-site cancer through a meta-analysis of 121 cohorts including 20 million individuals and one million events.[13] A 24-year follow-up study showed that depression increases the risk of cancer,[14] moreover, a meta-analysis of 16 studies (n=163,000) showed that cancer patients with anxiety and depression had a greater risk of dying from all types of cancer.[15] The research implied that the impairment of NK cell function is probably one of the common factors behind these links. For example, obesity has been known to impair NK cell function and then lead to an increased risk for severe infections and several cancer types,[16,17] while chronic family stress is consistently associated with decreases in NK cell cytotoxicity.[18] These results indicated that NK function impaired by either genetic defects or regulatory factors could increase cancer incidence.

eAppendix 3. Associations of the Inherited Defective Genes in APP and Wnt Pathways With Tumorigenesis and Metastasis

It has been shown that activated Wnt signaling pathway in tumors excluded the recruitment of CD8+ T cells into TIMEs.[19] Also, somatic mutations of the Wnt pathway in tumors could activate it to prevent T cells from being recruited into TIMEs.[20] Here we showed that in most of the cancer types the number of inherited defective genes in the Wnt pathway had a positive correlation with the gene expression of the Wnt pathway (i.e., pathway activation) in their paired tumors. In addition, the number of the inheritably defective genes in the Wnt pathway showed weakly negative correlations (i.e., with marginally significant p values in the range of 0.02-0.05) with the clinical outcomes for 8 cancer types (i.e., BLCA, BCRA, HNSC, LAUD, LUSC, PRAD, STAD and UCEC, eFigure 17). Also, the number of the inheritably defective genes in the Wnt pathway had negative correlations with the abundance of TILs in 6 cancer types (i.e., HNSC, KIRC, LGG, LUAD, LUSC and SKCM, eFigure 18) although the correlations were weaker (i.e., in terms of correlation co-efficiencies and correlation significance represented by p values) than

© 2019 Xu X et al. *JAMA Network Open.*

those derived from defective NK cells. These results suggested the association between inherited dysregulations in Wnt pathway and TILs' recruitment in some cancer types, and its influences were much weaker than defective NK cells.

APP is a biological process which presents antigens to T cells, functional defects in APP could lead tumor cells to escape T cell surveillance. However, careful analyses in this study showed that the number of genetic defective genes in the APP pathway (i.e., the KEGG APP pathway includes HLA family genes, TAP1/2 and other genes) was not significantly associated with clinical outcomes and TILs' abundance. However, significantly more inherited defective genes in the APP pathway were observed in cancer patients than cancer-free individuals in most cancers (Figure 5 in the main text). These results suggested that individuals who bear more inherited defective genes in the APP pathway probably were at-risk of developing cancers. These insights provided a potential opportunity to identify the subpopulation at-risk of developing cancers based on the inherited defective genes in NK cells and the APP pathway.

eAppendix 4. Open Questions Remained for NK Cell Inherited Defective Genes in Cancer

In this study, we showed that inherited defective genes in NK cells shaped TIME subtypes, TILs' abundance, survival and cancer risk. Along this line, many open questions still remained, for example, defective genes in NK cells were largely shared by different cancer types, however, each cancer type has a few unique NK-defective genes. Given the fact that tissue/organ-resident NK cells are different and diverse,[21] additional studies will be needed to elucidate if defective genes are dominantly expressed in tissue/organ-resident NK cells. If so, adoptive transfer of tissue-resident NK cells could be considered to be more efficient in cancer prevention and TIL's recruitment. NK cells share gene expression programs with NKT and γδ T cells, each of which is a subset of innate-like T cells. NK cells, γδ T cells and NKT cells are cytotoxic cells, which trigger innate immune responses, provide the first level of

© 2019 Xu X et al. *JAMA Network Open.*

defense against infected cells and tumor cells, produce cytokines and trigger immune responses without a prior sensitization by the immune system. Along with this point, we hypothesized that inherited defective genes in NKT and γδ T cells could also play important roles which were discussed in this study, although the cell number of the NK cells is nearly 200 times of that of the NKT and γδ T cells in periphery blood. Genetic defects in NK-NKT and NK-γδ T genes could impair the functions of not only NK cells but also NTK or γδ T cells, therefore, another possibility is that NK, NKT and γδ T cell impairment could contribute together to the results presented in this study. If this is the case, genome-editing to correcting the genetic defective genes in patients' hematopoietic stem cells which generate NK, NKT, γδ T and other immune cells could be an option for improving existing immunotherapies and cancer prevention for high-risk individuals.

© 2019 Xu X et al. *JAMA Network Open.*

eFigure 1. Heatmaps Showing the 3 Universal TIME Subtypes

Heatmaps showing the three universal TIME subtypes derived from the unsupervised clustering by using the expression of the immune-checkpoint therapy essential genes. Columns and rows represent samples and genes, respectively. Cancer types include bladder urothelial carcinoma (BLCA), breast invasive carcinoma (BRCA), colon adenocarcinoma (COAD), kidney renal clear cell carcinoma (KIRC), lower grade glioma (LGG), lung squamous cell carcinoma (LUSC), pancreatic adenocarcinoma (PRAD), skin cutaneous melanoma (SKCM), stomach adenocarcinoma (STAD), thyroid carcinoma (THCA) and uterine corpus endometrial carcinoma (UCEC). Red, green and blue bars represent TIME-rich, TIME-intermediate and TIME-poor subtype, respectively.

© 2019 Xu X et al. *JAMA Network Open.*

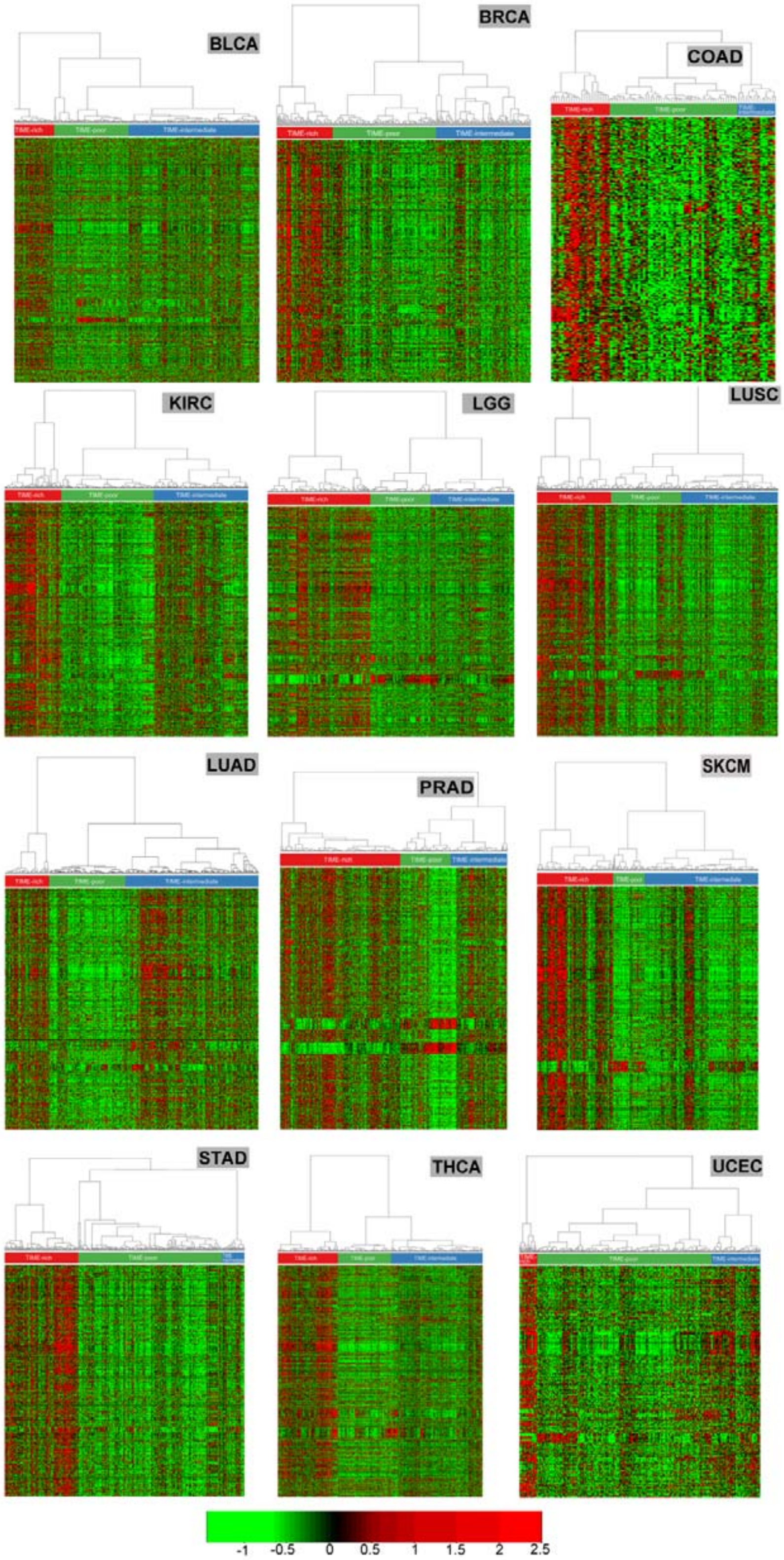

© 2019 Xu X et al. *JAMA Network Open.*

eFigure 2. Abundance of the Tumor-Infiltrating Lymphocytes in TIME Subtypes in Cancers
Rows represent tumor-infiltrating lymphocytes.

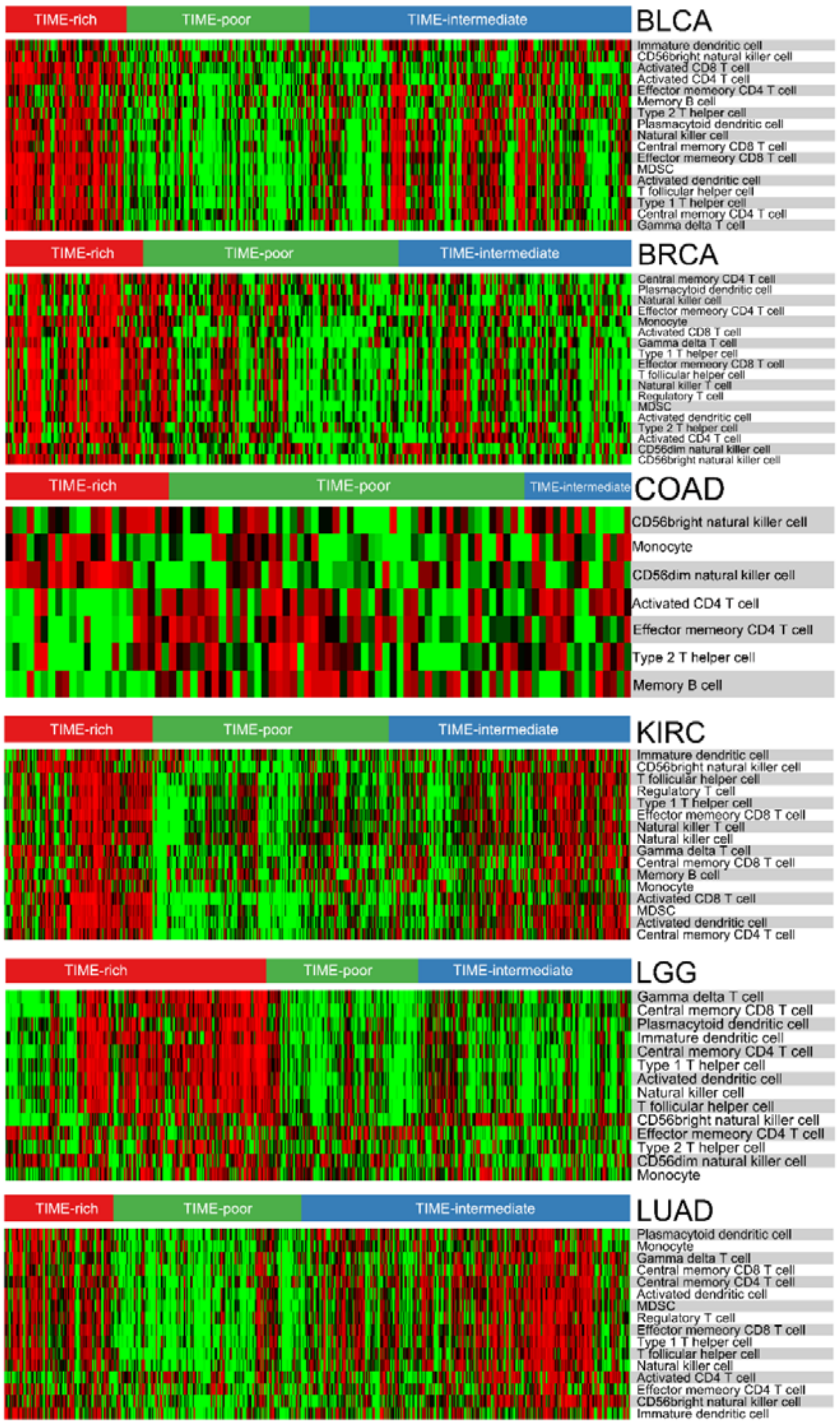

© 2019 Xu X et al. *JAMA Network Open.*

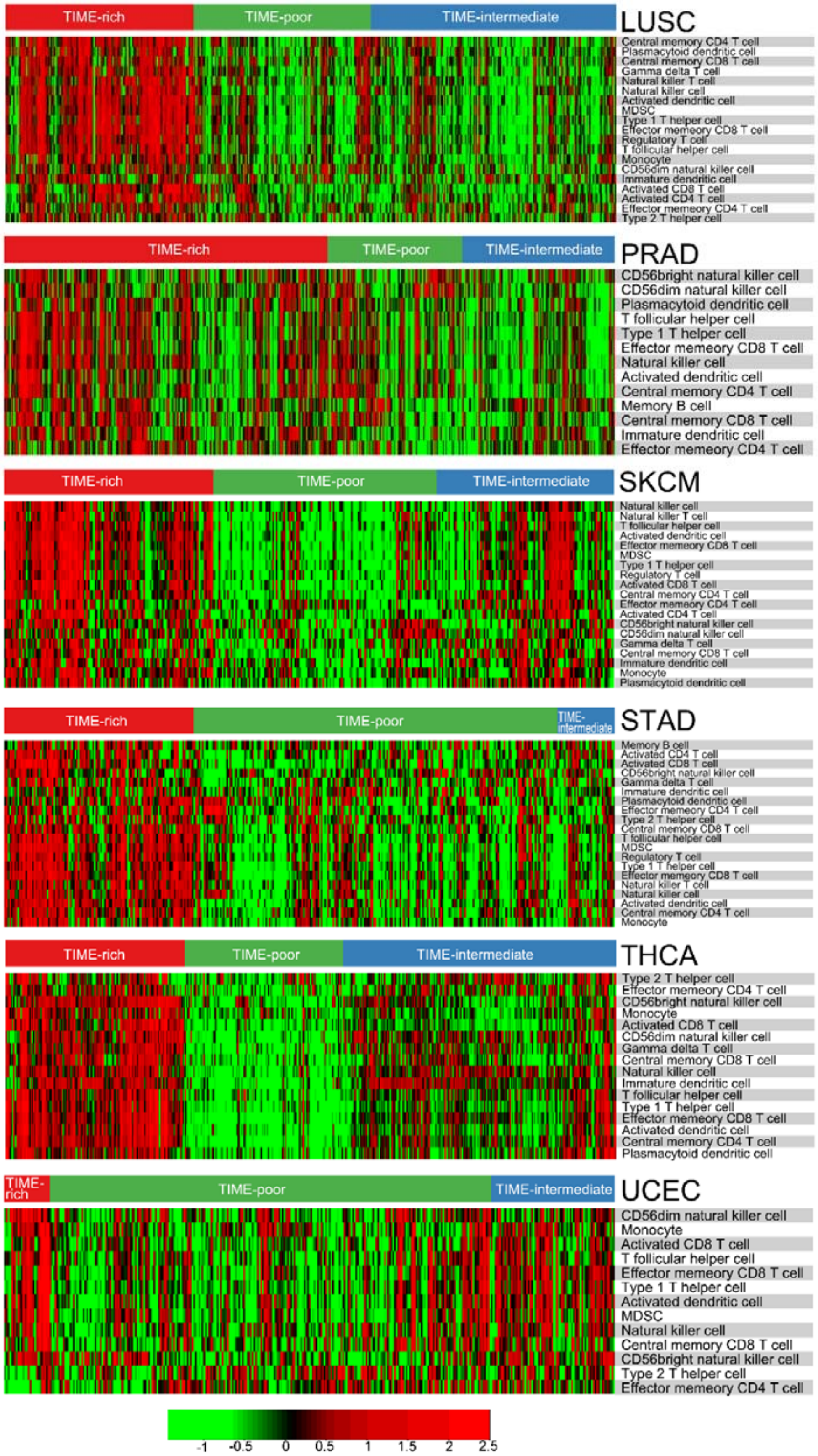

© 2019 Xu X et al. *JAMA Network Open.*

eFigure 3. Kaplan-Meier Curves Between Patients With Cancer in TIME-Rich Subtype and TIME-Intermediate and TIME-Poor Subtypes.
It revealed that survival for was significantly longer for TIME-rich patients than TIME-intermediate/-poor patients. A substantial fraction of samples in COAD was virus-infected tumors After removing them, only 87 COAD samples were remained for analysis.

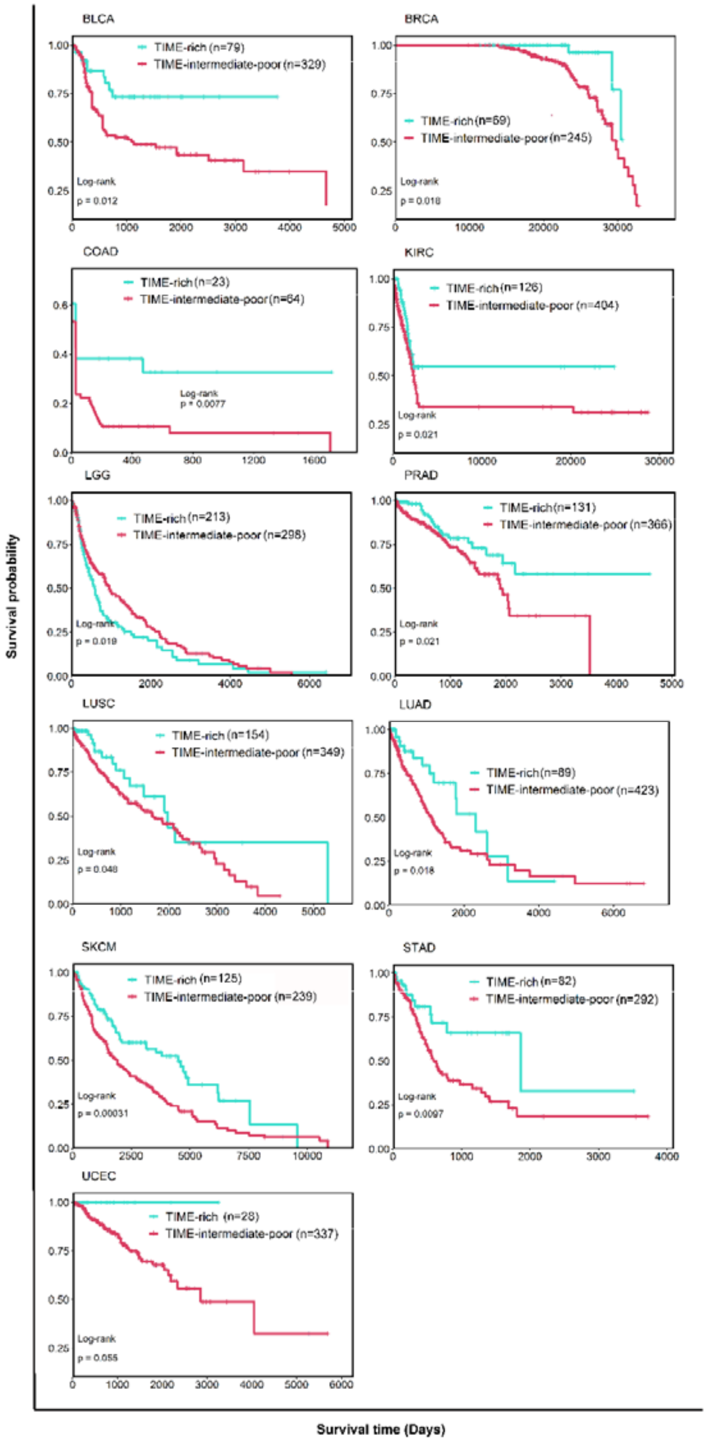

© 2019 Xu X et al. *JAMA Network Open.*

eFigure 4. Significantly Enriched Pathways by Comparing RNA-seq Data in TIME-Intermediate and TIME-Poor Subtypes

Columns and rows represent KEGG pathways and cancer types, respectively. The digital numbers represent FDR-corrected p values.

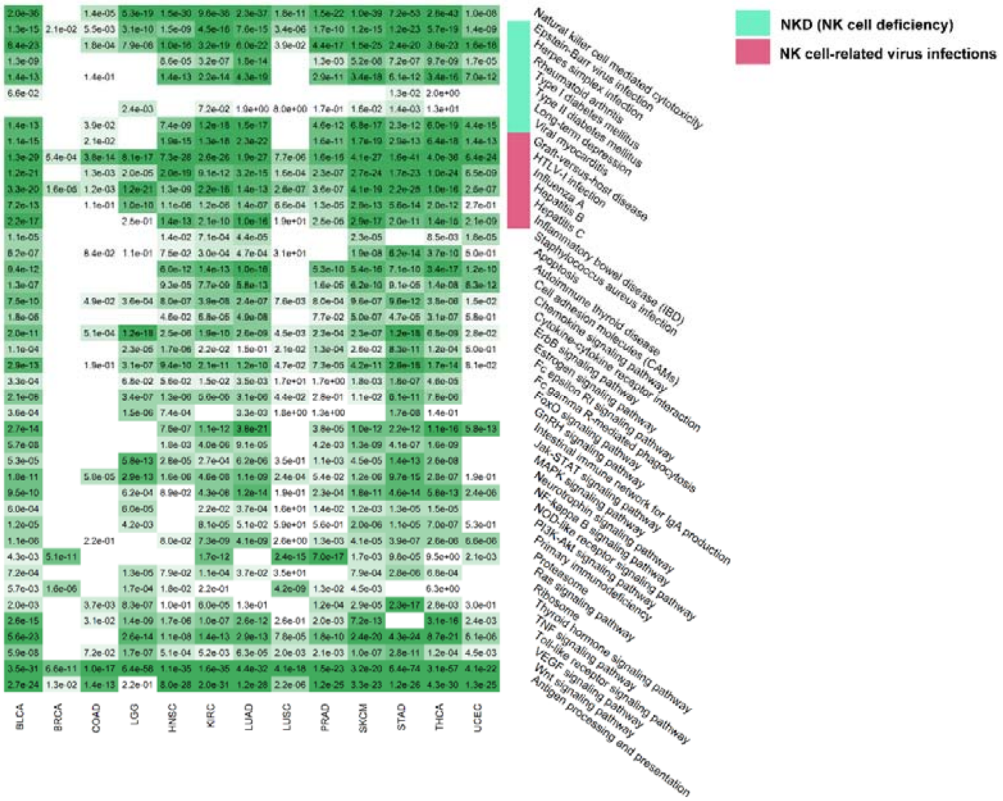

© 2019 Xu X et al. *JAMA Network Open.*

eFigure 5. Heatmaps of the Significantly Differential Functional Germline Variants Between the TIME-Rich and TIME-Intermediate/TIME-Poor Subtypes
(Fisher's exact tests with FDR-corrected p<0.25). Only the functional germline variants (rows) which were less enriched in the TIME-rich subtype are shown. Red, green and blue bars represent TIME-rich, TIME-intermediate and TIME-poor subtype, respectively. Columns represent samples.

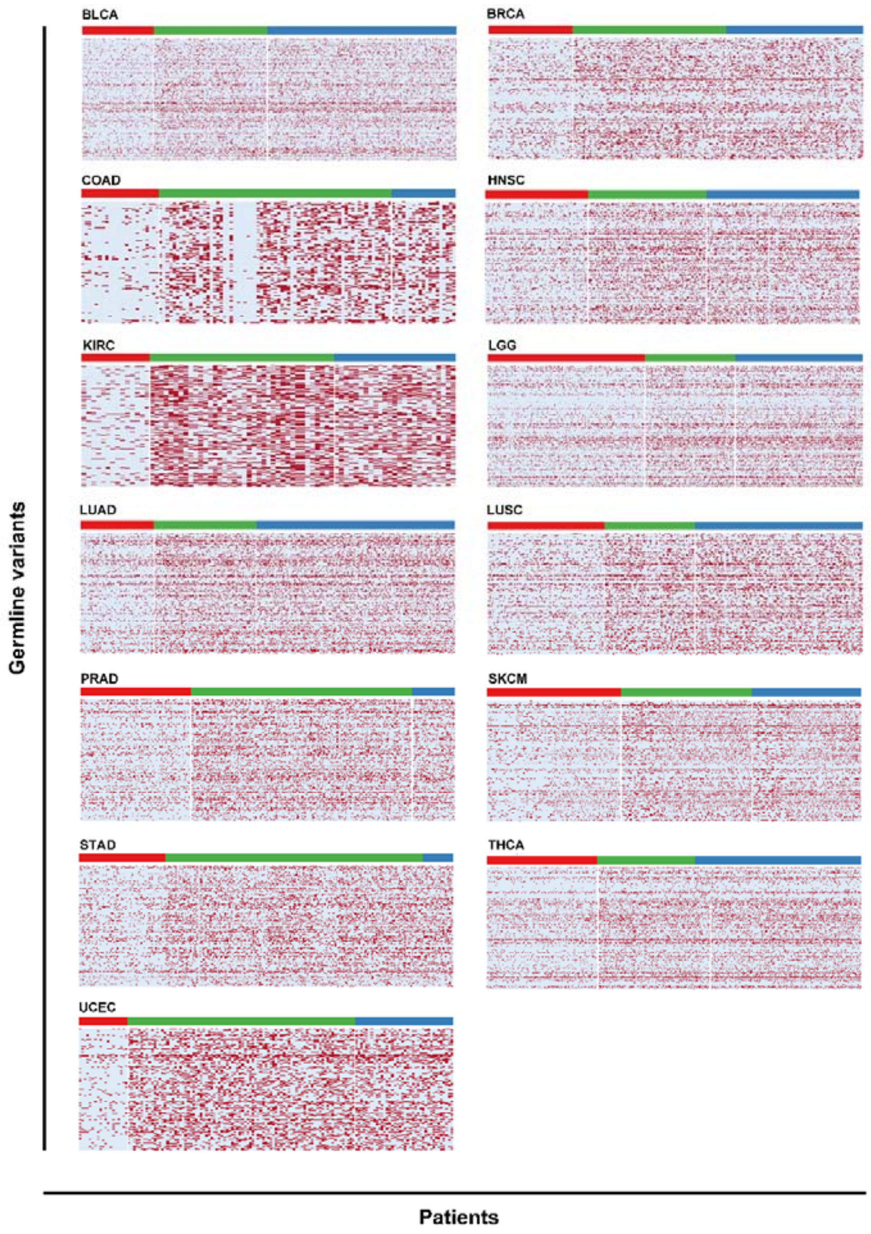

© 2019 Xu X et al. *JAMA Network Open.*

eFigure 6. Significantly Enriched Pathways by Comparing Functional Germline Variant Between TIME-Rich and TIME-Intermediate/TIME-Poor Subtypes

Columns and rows represent KEGG pathways and cancer types, respectively. The digital numbers represent FDR-corrected p values. Significantly functional differential germline variants (p<0.2) between TIME-rich and TIME-intermediate/-poor tumors were intersected with the immune-checkpoint therapy (ICT) essential genes, and then pathway enrichment analysis was conducted.

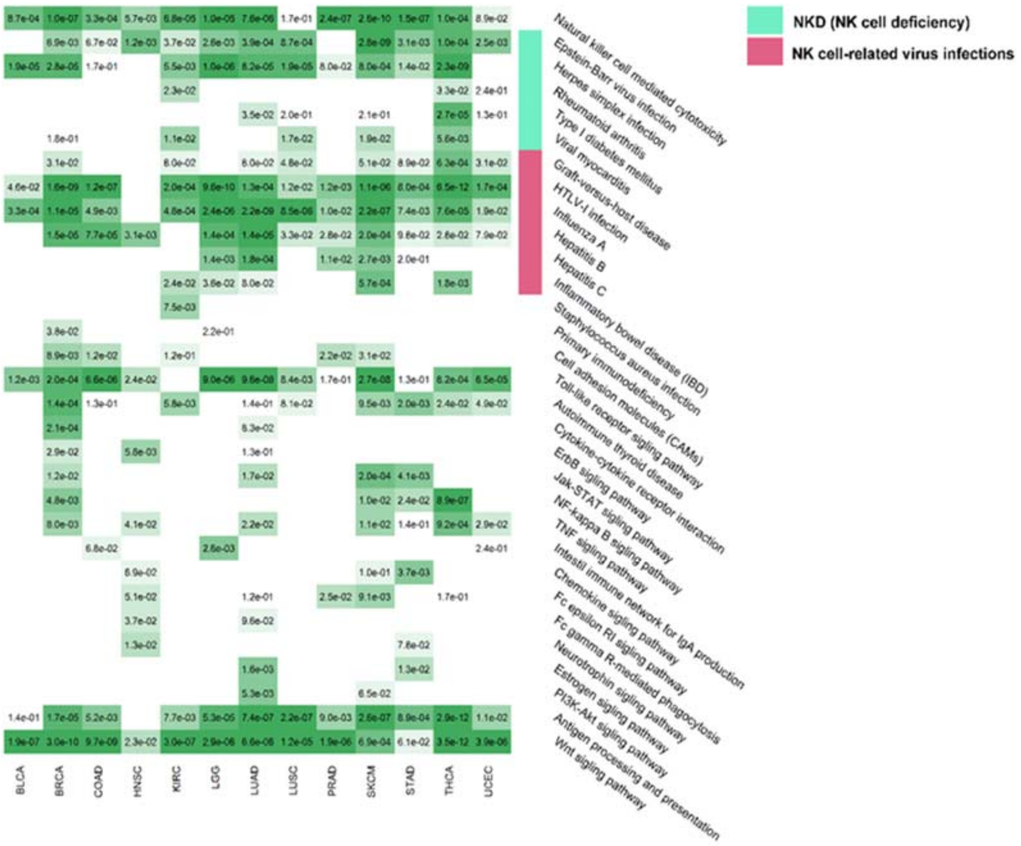

© 2019 Xu X et al. *JAMA Network Open.*

eFigure 7. Significantly Enriched Pathways of the Significantly Differential Germline Variants Between the TIME-Intermediate and TIME-Poor Subtypes
A heatmap showing the significantly enriched pathways derived from the significantly differential germline variants between TIME-intermediate and TIME-poor subtypes. Columns and rows represent KEGG pathways and cancer types, respectively. The digital numbers represent FDR-corrected p values. Significantly functional differential germline variants (p<0.2) between TIME-intermediate and TIME-poor tumors were intersected with the immune-checkpoint therapy (ICT) essential genes, and then pathway enrichment analysis was conducted.

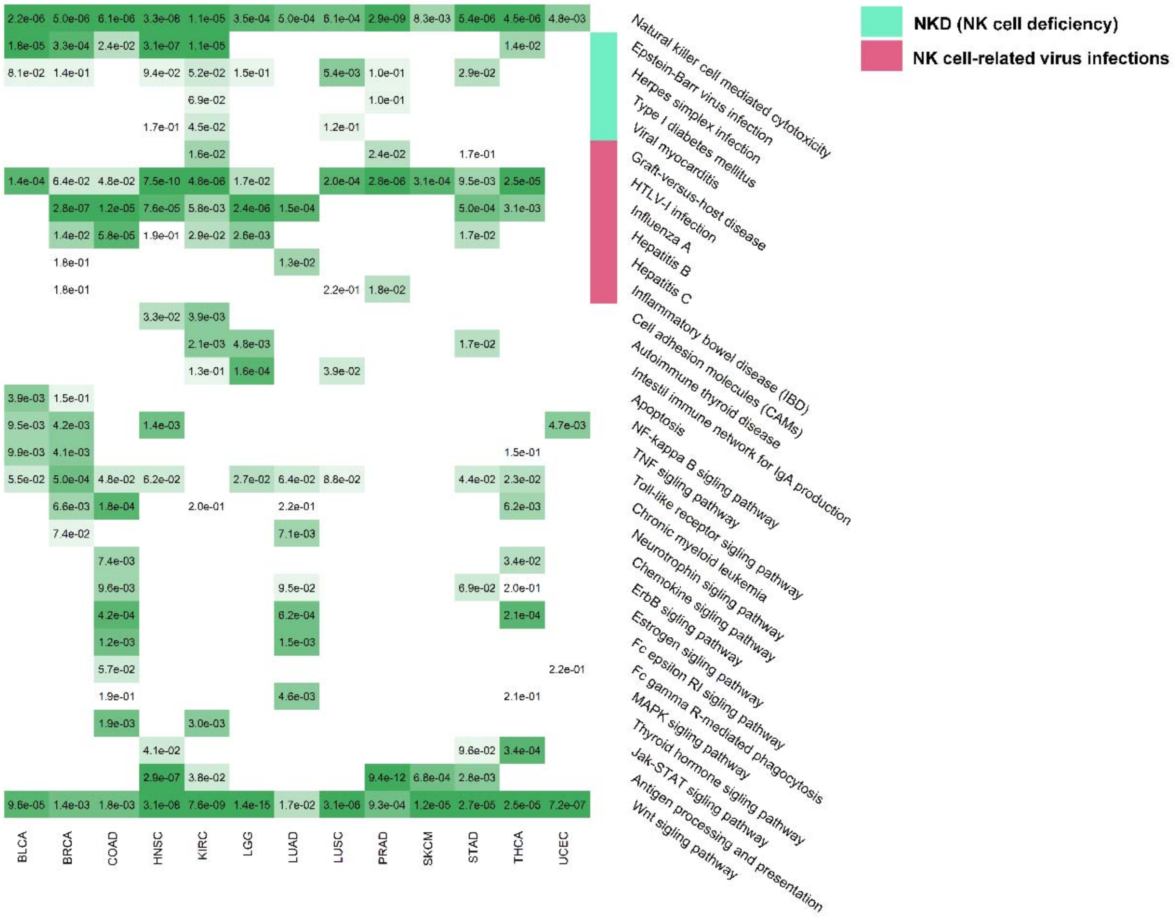

© 2019 Xu X et al. *JAMA Network Open.*

eFigure 8. A Heatmap Showing the Significantly More Inherited NKD Genes in TIME-Intermediate/TIME-Poor Subtypes Than TIME-Rich Subtype in Cancers

The digital numbers represent FDR-corrected p values. Columns and rows represent cancer types and known inherited NKD (NK cell deficiency) genes, respectively.

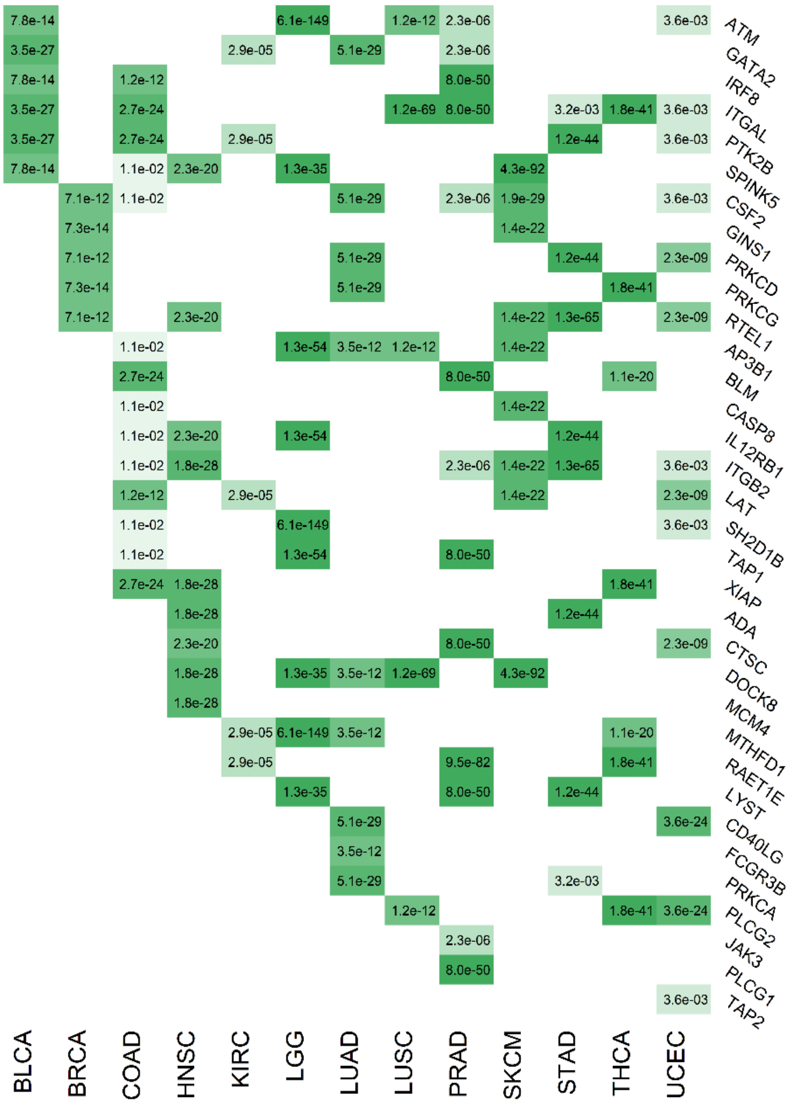

© 2019 Xu X et al. *JAMA Network Open.*

eFigure 9. A Bar Chart Showing Ratios of Gene Categories of the Potential NKD Genes Across 12 Cancer Types

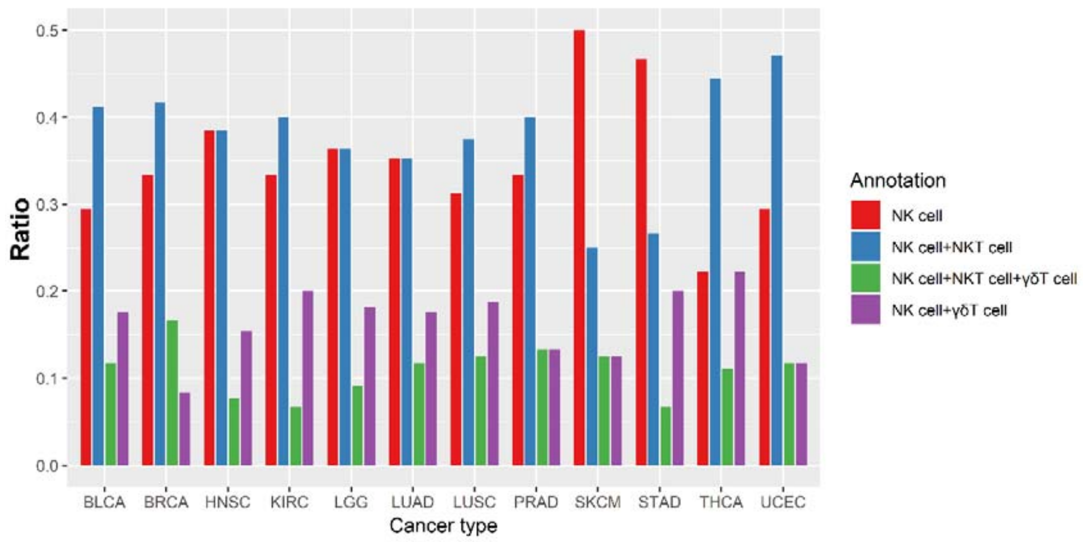

© 2019 Xu X et al. *JAMA Network Open.*

eFigure 10. Kaplan-Meier Curves of the High- and Low-Number of Functionally Inherited NK Cell Variants

Kaplan-Meier curves of the patient groups of the high- and low-number of functionally inherited variants in the NK-defective genes for disease-free survival in cancers. Patients were top-ranked based on the number of functionally inherited variants in NK cells. Top 30% and Bottom 30% of the ranked patients were defined as the high- and low-number of the NK-defective patient groups, respectively.

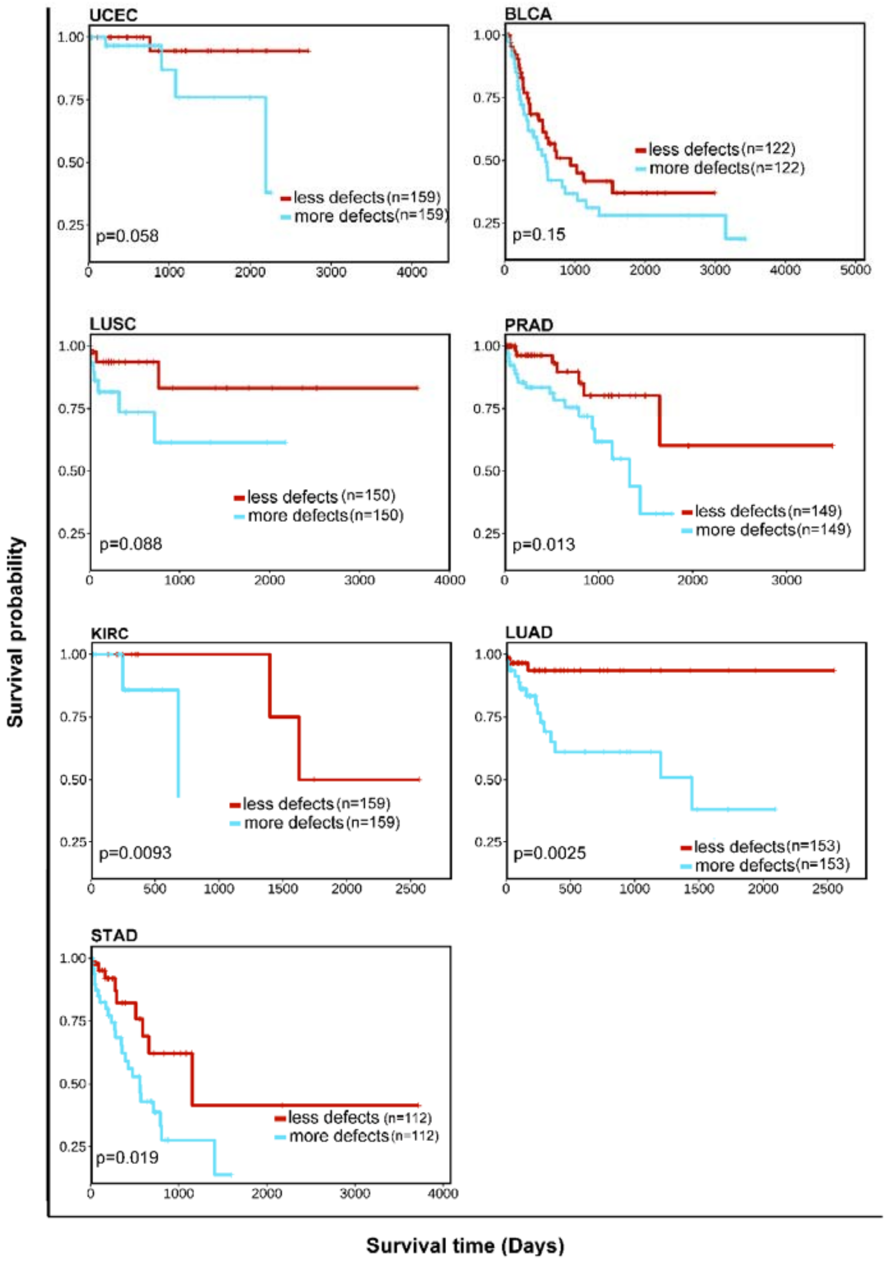

© 2019 Xu X et al. *JAMA Network Open.*

eFigure 11. Negative Correlations Between the Number of the Inheritable Defective Genes and Abundance of TILs

NK, NKT, A CD8+ T, A CD4+ T, cDC, immature B, Activated B, MDSC, GD T, RT, CM CD8+ T, I dendritic, Type 1 TH, Type 2 TH, Type 17 TH, TF helper, A dendritic, EM CD4+ T, EM CD8+ T, P dendritic and CM CD4+ T represent natural killer cell, natural killer T cell, activated CD8+ T cell, activated CD4+ T cell, conventional dendritic cell, immature B cell, activated B cell, myeloid-derived suppressor cell, gamma delta T cell, regulatory T cell, central memory CD8+ T cell, immature dendritic cell, Type 1 T helper cell, Type 2 T helper cell, Type 17 T helper cell, T follicular helper cell, activated dendritic cell, effector memory CD4+ T cell, effector memory CD8+ T cell, plasmacytoid dendritic cell and central memory CD4+ T cell, respectively. *0.05<p-value <0.1; **0.01<p-value <0.05; and ***p-value<0.01.

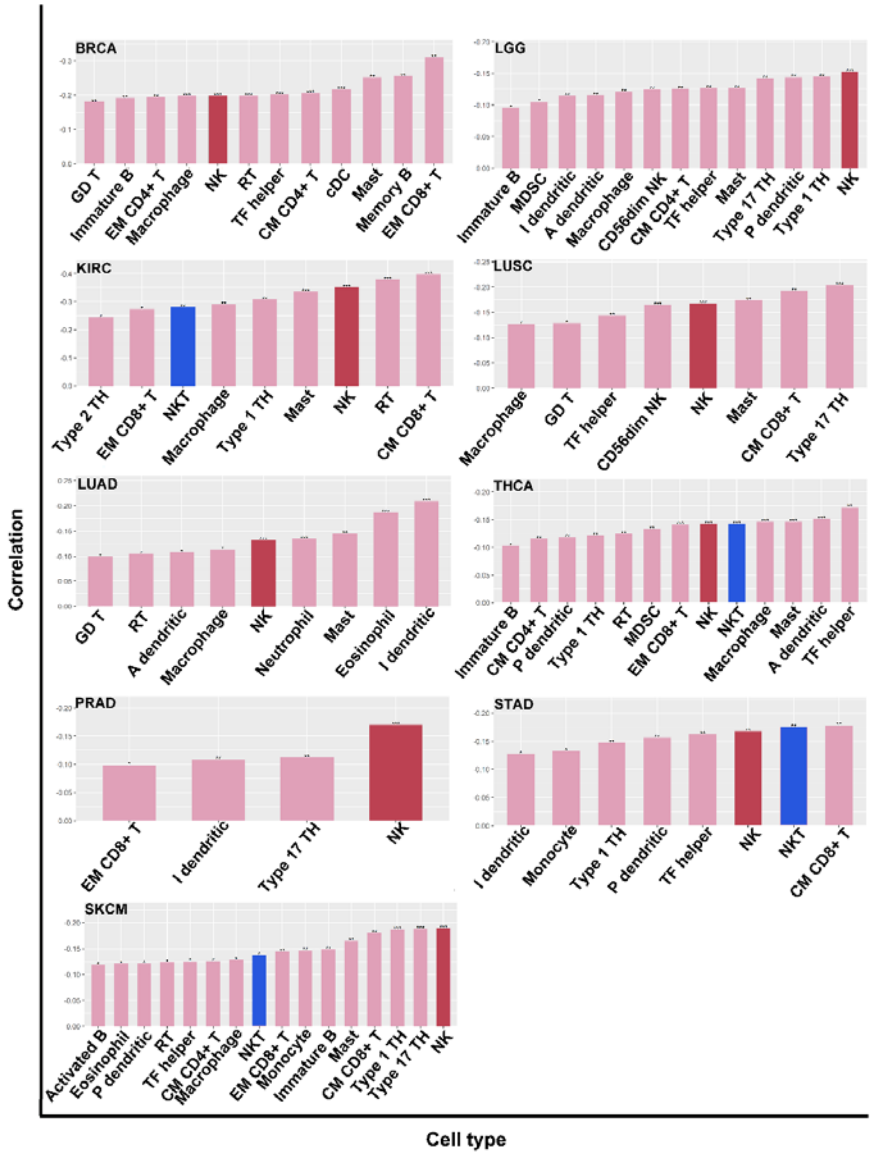

© 2019 Xu X et al. *JAMA Network Open.*

eFigure 12. Kaplan-Meier Curves of the High- and Low-Number of Functionally Inherited Variants of the Combined Genes

Kaplan-Meier curves of the patient groups of the high- and low-number of merged functionally inherited variants in the combined genes (NK-defective genes + defective non-NK-specific ITAM-signaling genes) for disease-free survival in cancers. Patients were top-ranked based on the number of the functionally inherited variants. Top 30% and Bottom 30% of the ranked patients were defined as the high- and low-number of the defective patient groups, respectively.

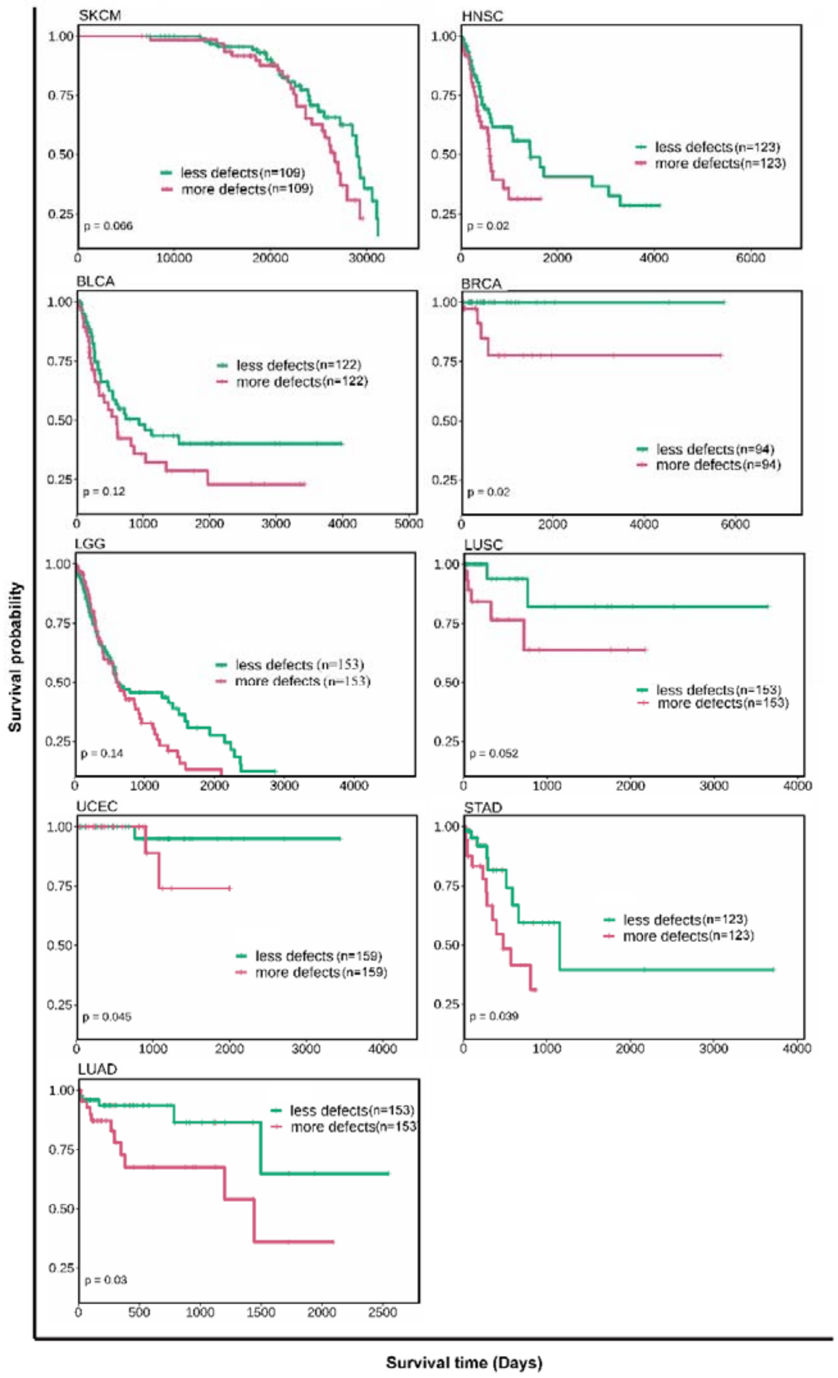

© 2019 Xu X et al. *JAMA Network Open.*

eFigure 13. Negative Correlations Between the Number of the Inheritable Defective Combined Genes and Abundance of TILs

Negative correlations between the number of the inheritable defective genes of the combined genes (NK-defective genes + defective non-NK-specific ITAM-signaling genes) and the abundance of tumor-infiltrating lymphocytes in cancers. NK, NKT, A CD8+ T, A CD4+ T, cDC, immature B, Activated B, MDSC, GD T, RT, CM CD8+ T, I dendritic, Type 1 TH, Type 2 TH, Type 17 TH, TF helper, A dendritic, EM CD4+ T, EM CD8+ T, P dendritic and CM CD4+ T represent natural killer cell, natural killer T cell, activated CD8+ T cell, activated CD4+ T cell, conventional dendritic cell, immature B cell, activated B cell, myeloid-derived suppressor cell, gamma delta T cell, regulatory T cell, central memory CD8+ T cell, immature dendritic cell, Type 1 T helper cell, Type 2 T helper cell, Type 17 T helper cell, T follicular helper cell, activated dendritic cell, effector memory CD4+ T cell, effector memory CD8+ T cell, plasmacytoid dendritic cell and central memory CD4+ T cell, respectively. *0.05<p-value <0.1; **0.01<p-value <0.05; and ***p-value<0.01.

© 2019 Xu X et al. *JAMA Network Open.*

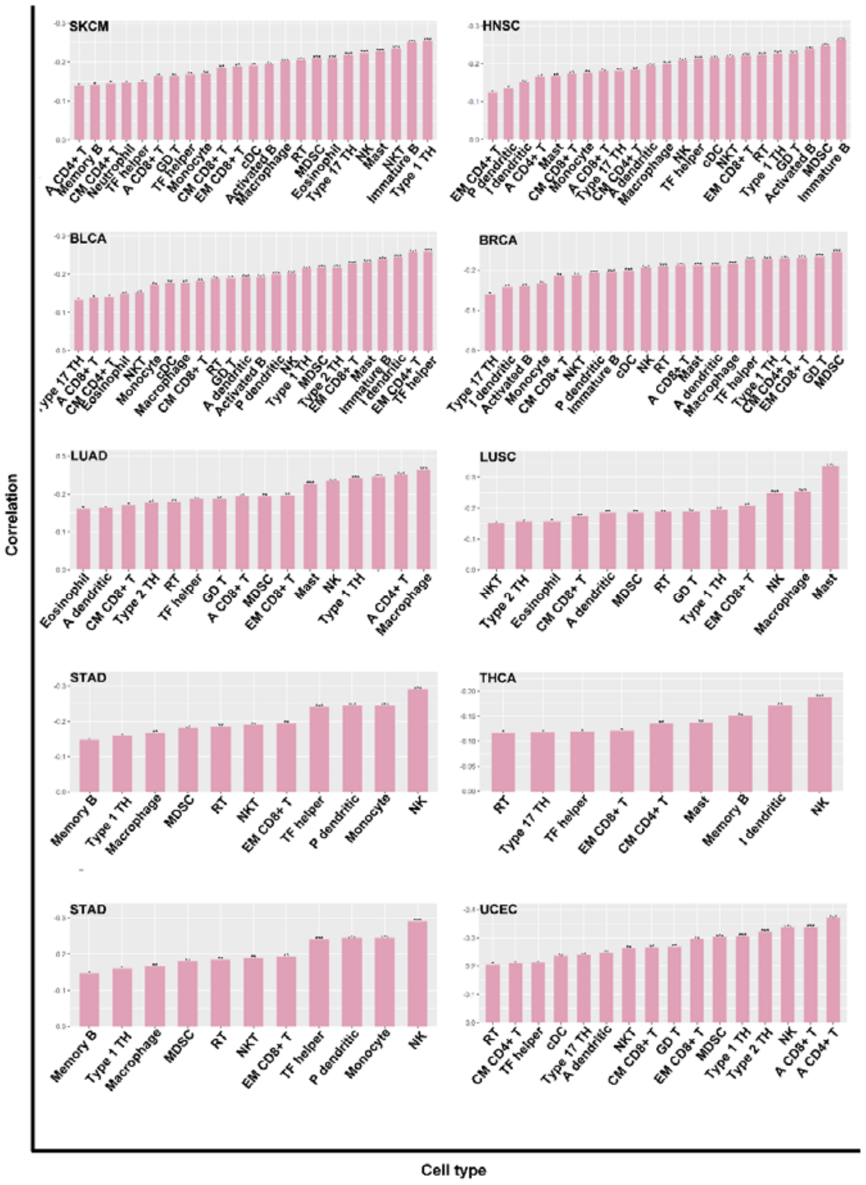

© 2019 Xu X et al. *JAMA Network Open.*

eFigure 14. The Abundance of TIL-NK Cells in the Tumors Bearing a Defective Gene in NK Cells Was Significantly Lower Than the Rest of the Tumors
T-tests were conducted.

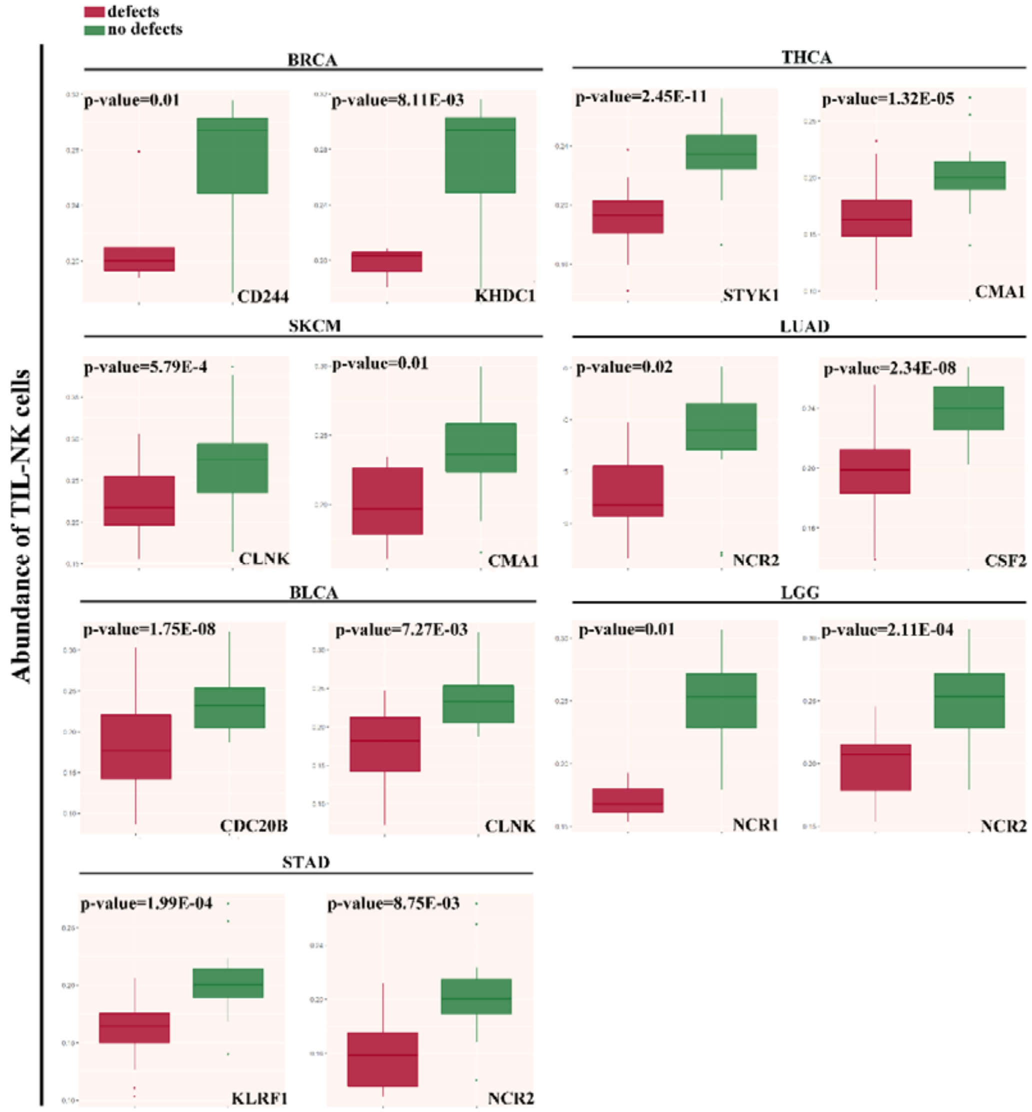

© 2019 Xu X et al. *JAMA Network Open.*

eFigure 15. Heatmaps of the Significantly Differentially Functional Germline Variants Between Cancer and Cancer-Free Cohorts
Only the functional germline variants (rows) which are more enriched in cancer patients are shown. For each cancer types, the same number of the cancer-free individuals were randomly selected from the cancer-free cohort (n=4,500). Red and blue bars represent cancer and cancer-free individuals, respectively. Columns represent samples.

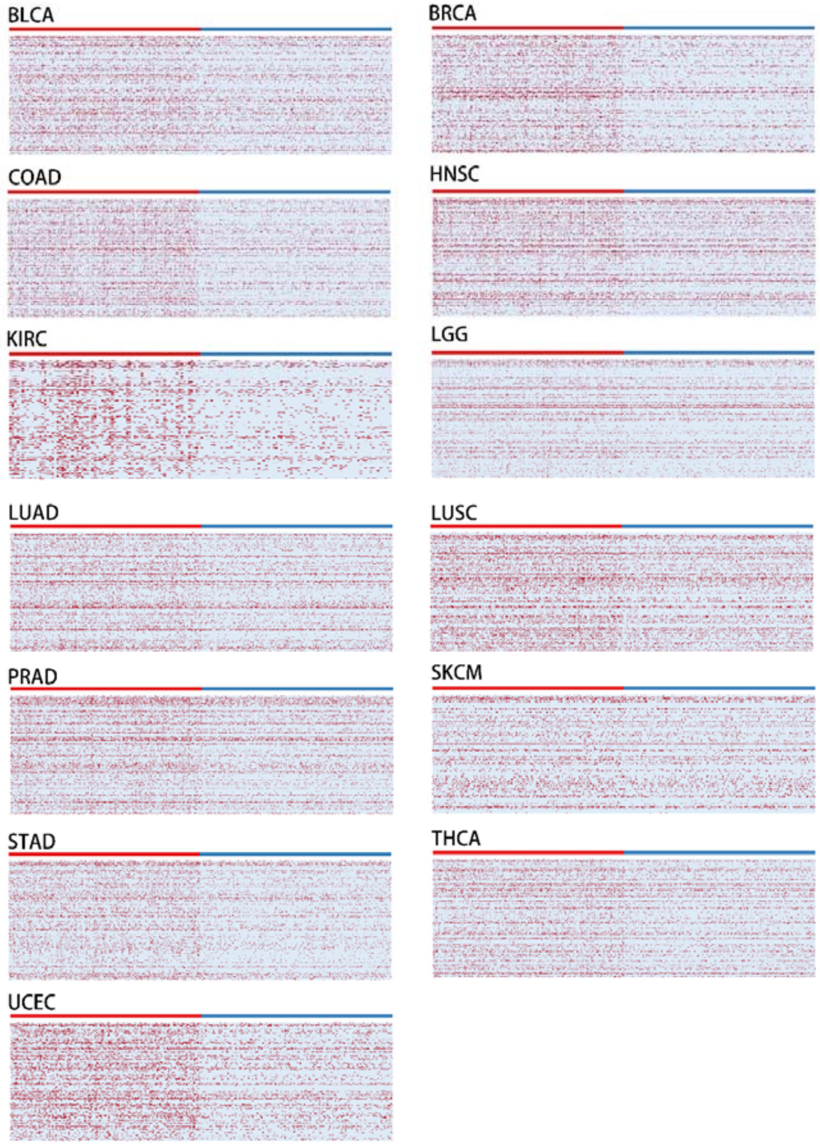

© 2019 Xu X et al. *JAMA Network Open.*

eFigure 16. Significantly Enriched Pathways Derived from the Significantly Differential Germline Variants Between Individuals With No Cancer Patients With Cance rA heatmap showing the significantly enriched pathways derived from the significantly differential germline variants between cancer-free individuals and cancer patients in 13 cancer types. Columns and rows represent KEGG pathways and cancer types, respectively. The digital numbers represent FDR-corrected p values. Significantly functional differential germline variants (p<0.2) between cancer-free individuals and cancer patients were intersected with the immune-checkpoint therapy (ICT) essential genes, and then pathway enrichment analysis was conducted.

	BLCA	BRCA	COAD	HNSC	KIRC	LGG	LUAD	LUSC	PRAD	SKCM	STAD	THCA	UCEC
Natural killer cell mediated cytotoxicity	2.6e-20	4.6e-23	1.9e-20	9.2e-28	6.8e-37	1.5e-32	2.2e-27	1.4e-13	2.5e-25	2.6e-28	3.5e-22	3.1e-33	6.8e-37
Epstein-Barr virus infection	1.2e-13	3.0e-14	3.7e-08	1.4e-13	3.4e-09	1.4e-13	4.0e-08	1.1e-02	2.9e-13	2.1e-07	1.4e-08	2.2e-16	5.9e-15
Herpes simplex infection	2.0e-13	2.1e-14	6.5e-07	5.7e-17	3.4e-11	2.4e-12	3.9e-09		5.7e-13	3.6e-08	3.5e-09	6.2e-20	3.7e-14
Rheumatoid arthritis	2.3e-04	6.1e-02		2.4e-04		4.9e-04			1.2e-01		1.4e-03	3.0e-03	
Type I diabetes mellitus		1.8e-05	1.1e-03	1.3e-10		1.1e-07	3.9e-02		8.0e-06		3.4e-06	6.6e-08	2.9e-06
Long-term depression			2.4e-01										1.1e-01
Viral myocarditis		1.3e-03	2.7e-02	8.4e-08		2.9e-08	4.2e-03		8.4e-05	1.8e-01	1.1e-08	2.5e-07	1.5e-07
Graft-versus-host disease	5.0e-04	5.6e-06	7.2e-05	2.9e-13	4.6e-03	1.2e-09	3.7e-03		2.6e-06		3.6e-09	4.9e-10	3.4e-08
HTLV-I infection	3.4e-01	1.3e-19	1.4e-09	6.3e-22	4.7e-16	2.5e-19	1.2e-17	1.5e-08	4.5e-17	4.4e-10	1.7e-22	3.7e-21	2.3e-15
Influenza A	3.7e-11	2.7e-11	1.2e-09	1.0e-19	4.1e-15	7.4e-11	1.5e-10		5.7e-13	1.0e-08	2.4e-11	1.9e-18	5.2e-20
Hepatitis B	1.4e-11	1.2e-11	2.6e-10	1.0e-12	3.7e-16	2.9e-16	4.2e-14		7.5e-11	4.7e-09	9.0e-12	1.9e-21	9.4e-19
Hepatitis C	4.1e-08	4.1e-08	1.9e-01	5.3e-07	2.0e-07	1.6e-05	1.7e-06		1.4e-06	3.9e-05	1.4e-02	1.0e-10	2.7e-10
Inflammatory bowel disease (IBD)	5.6e-03	5.6e-03	1.6e-02	1.1e-08	5.1e-02	2.3e-02	8.1e-02		1.5e-02		3.1e-04	1.1e-04	2.3e-03
Primary immunodeficiency	6.7e-05	9.6e-05		1.1e-02		5.0e-02		2.5e-02	3.6e-02	2.2e-02	1.2e-02	3.0e-05	6.4e-03
Autoimmune thyroid disease		4.6e-11	2.5e-07	6.0e-18	2.1e-09	2.1e-12	3.0e-07		8.5e-13	2.2e-05	9.4e-12	3.3e-17	3.7e-16
Cell adhesion molecules (CAMs)		9.7e-05	3.6e-02	2.6e-06	1.3e-02	1.4e-05	5.9e-04		2.8e-05		7.0e-07	1.8e-04	2.1e-05
Intestil immune network for IgA production	1.5e-05	1.1e-05		1.5e-09	1.9e-05	7.8e-06	1.9e-02		4.5e-06		1.7e-04	5.0e-06	1.9e-05
FoxO sigling pathway		2.7e-02			4.7e-02	2.1e-01		1.0e-02	1.3e-01			1.1e-02	
NOD-like receptor sigling pathway		3.4e-02		2.4e-01		2.7e-02					1.8e-03	8.5e-05	
Thyroid hormone sigling pathway		1.3e-01	7.3e-05	2.0e-01	5.6e-05	9.6e-02			1.5e-02		1.3e-03	1.8e-04	3.7e-03
GnRH sigling pathway			4.3e-05		4.9e-05	1.7e-02							9.6e-06
Neurotrophin sigling pathway			7.2e-04	3.1e-02	1.4e-04	4.8e-02			2.8e-02	6.2e-06	1.2e-01	1.7e-03	3.3e-05
VEGF sigling pathway			5.3e-02		2.9e-02	2.3e-03	2.4e-01	1.8e-01		2.0e-05	2.8e-02	1.0e-02	1.1e-03
Ras sigling pathway										1.6e-01		6.0e-02	1.3e-01
Antigen processing and presentation	5.5e-10	5.9e-21	3.5e-10	8.5e-15	2.7e-08	6.7e-19	1.1e-09	8.2e-03	6.7e-15	9.2e-10	1.9e-21	2.4e-18	2.7e-19
Wnt sigling pathway	7.0e-14	5.7e-15	2.0e-15	2.8e-15	5.2e-33	5.8e-20	1.6e-20	8.4e-13	1.1e-19	2.5e-11	1.0e-26	1.4e-26	1.4e-24

© 2019 Xu X et al. *JAMA Network Open.*

eFigure 17. Kaplan-Meier Curves of the High- and Low-Number of Functionally Inherited Variants in the Wnt Signaling Pathway for Disease-Free Survival

Patients were top-to-bottom ranked based on the number of functionally inherited variants in the WNT signaling pathway. Top 30% and Bottom 30% of the ranked patients were defined as the high- and low-number of the defected defects in the pathway, respectively.

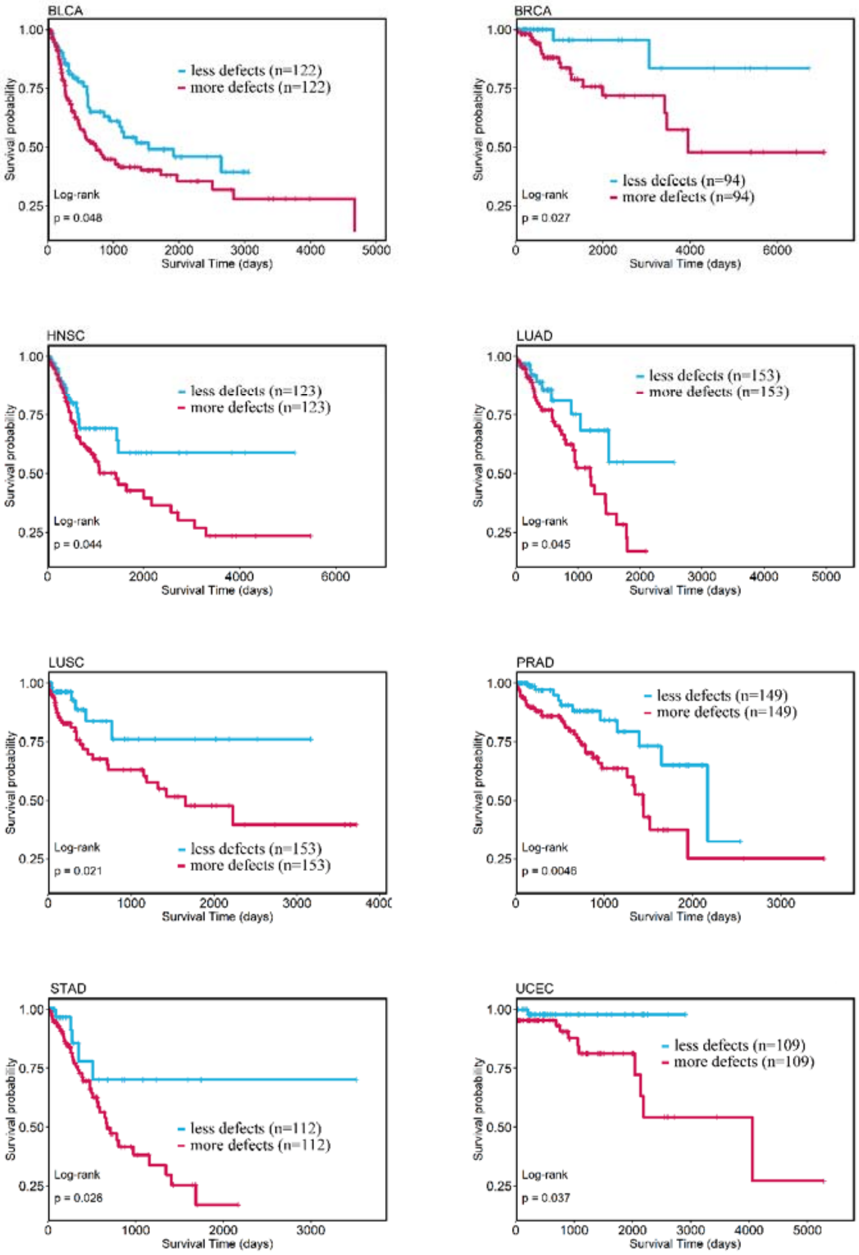

© 2019 Xu X et al. *JAMA Network Open.*

eFigure 18. Correlations of the Functionally Inherited Variants in the Wnt Signaling Pathway With the Abundance of TILs

Bar charts illustrating the number of the functionally inherited variants (inherited defects) in the Wnt signaling pathway is negatively correlated with the abundances of tumor-infiltrating lymphocytes. Cancer types: head and neck squamous cell carcinoma (HNSC), kidney renal clear cell carcinoma (KIRC), lower grade glioma (LGG), lung adenocarcinoma (LUAD), lung squamous cell carcinoma (LUSC) and skin cutaneous melanoma (SKCM). * p<0.05; ** 0.05<p>0.001 and *** p<0.001.

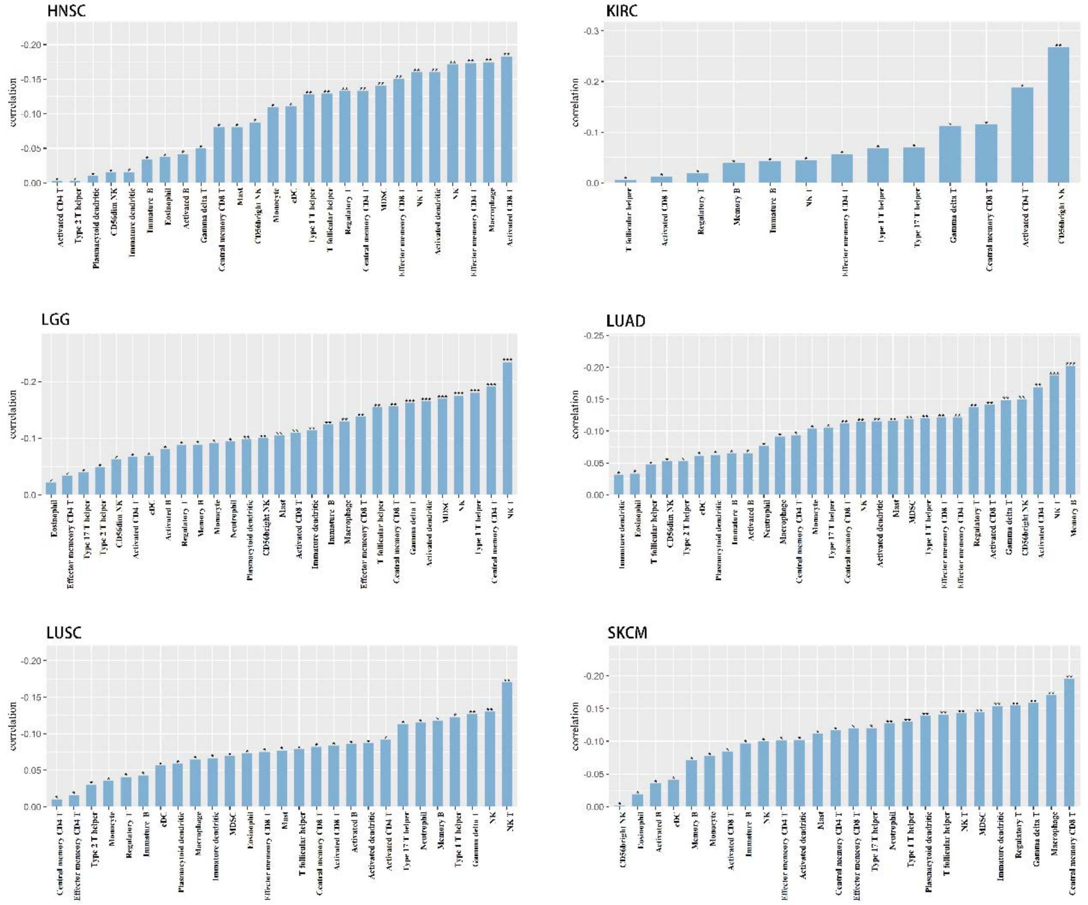

© 2019 Xu X et al. *JAMA Network Open.*

eTable 1. List of ITAM-Signaling Genes

ITAM-signaling genes
ULBP1
ULBP2
ULBP3
MICA
MICB
CD94
NKG2A
NKG2C
HLA-E
CD16
DNAM-1
CD226
ITGB2
ICAM1
ZAP70
PLCG1
VAV1
PLCG2

© 2019 Xu X et al. *JAMA Network Open.*

eTable 2. List of Ligands of the NK Activating Receptors

**Ligands of NK cell activating receptors**
TNFRSF14
PVR
NECTIN2
CD48
CADM1
HLA-E
MICA
MICB
RAET1E
RAET1G
RAET1F
ULBP1
ULBP2
ULBP3
RAET1L
VIM
KMT2E
PCNA
PDGFD
BAG6
NCR3LG1
TNFSF9
CD274
PDCD1LG2
FAS
FASLG

© 2019 Xu X et al. *JAMA Network Open.*

TNFRSF10B
TNFRSF10A
ICAM1
CSF2
CSF1
CSF3
CD70
SELL
CD72
CLEC2B
CEACAM1
SLAMF6
SLAMF7

© 2019 Xu X et al. *JAMA Network Open.*

eTable 3. Average Immune Cell Fractions for TIME-Rich, TIME-Intermediate and TIME-Poor Subtypes, Respectively

**Cancer**	**Immune cell**	**TIME-rich**	**TIME-intermediate**	**TIME-poor**	**P-value**
BLCA	Activated CD4+ T cell	0.25	0.18	0.14	1.22E-19
BLCA	Activated CD8+ T cell	0.26	0.14	0.14	9.75E-19
BLCA	Activated dendritic cell	0.26	0.21	0.17	6.37E-27
BLCA	$CD56^{bright}$ natural killer cell	0.27	0.25	0.25	7.75E-09
BLCA	$CD56^{dim}$ natural killer cell	0.33	0.33	0.34	4.73E-01
BLCA	Central memory CD4+ T cell	0.55	0.52	0.50	2.24E-13
BLCA	Central memory CD8+ T cell	0.35	0.33	0.30	5.10E-08
BLCA	Effector memory CD4+ T cell	0.16	0.15	0.13	3.12E-03
BLCA	Effector memory CD8+ T cell	0.32	0.20	0.15	4.70E-31
BLCA	Gamma delta T cell	0.29	0.25	0.24	4.65E-16
BLCA	Immature dendritic cell	0.35	0.34	0.33	5.46E-04
BLCA	MDSC	0.36	0.20	0.13	2.30E-20
BLCA	Memory B cell	0.10	0.09	0.07	3.07E-03
BLCA	Monocyte	0.36	0.36	0.36	6.73E-01
BLCA	Natural killer cell	0.28	0.24	0.20	7.85E-17
BLCA	Plasmacytoid dendritic cell	0.38	0.36	0.34	3.59E-07

© 2019 Xu X et al. *JAMA Network Open.*

BLCA	T follicular helper cell	0.18	0.13	0.10	5.61E-14
BLCA	Type 1 T helper cell	0.17	0.12	0.09	1.53E-16
BLCA	Type 2 T helper cell	0.15	0.13	0.10	3.66E-12
BRCA	Activated CD4+ T cell	0.20	0.14	0.10	5.66E-09
BRCA	Activated CD8+ T cell	0.24	0.14	0.12	3.11E-13
BRCA	Activated dendritic cell	0.25	0.20	0.20	9.72E-15
BRCA	$CD56^{bright}$ natural killer cell	0.25	0.22	0.22	1.23E-13
BRCA	$CD56^{dim}$ natural killer cell	0.35	0.33	0.32	7.54E-04
BRCA	Central memory CD4+ T cell	0.54	0.51	0.51	6.63E-08
BRCA	Central memory CD8+ T cell	0.35	0.34	0.35	2.56E-01
BRCA	Effector memory CD4+ T cell	0.16	0.15	0.15	3.61E-02
BRCA	Effector memory CD8+ T cell	0.29	0.20	0.20	4.10E-11
BRCA	Gamma delta T cell	0.25	0.23	0.22	1.55E-07
BRCA	Immature dendritic cell	0.34	0.33	0.34	1.58E-01
BRCA	MDSC	0.34	0.22	0.20	7.81E-13
BRCA	Memory B cell	0.13	0.14	0.13	7.10E-01
BRCA	Monocyte	0.36	0.33	0.33	4.11E-10
BRCA	Natural killer cell	0.29	0.26	0.27	2.65E-04
BRCA	Natural killer T cell	0.09	0.05	0.05	4.08E-10
BRCA	Plasmacytoid dendritic cell	0.40	0.38	0.39	4.96E-04

© 2019 Xu X et al. *JAMA Network Open.*

BRCA	Regulatory T cell	0.20	0.12	0.12	1.77E-09
BRCA	T follicular helper cell	0.16	0.12	0.11	5.60E-09
BRCA	Type 1 T helper cell	0.17	0.12	0.12	1.19E-09
BRCA	Type 2 T helper cell	0.16	0.13	0.13	3.51E-03
COAD	Activated CD4+ T cell	0.10	0.21	0.19	8.52E-03
COAD	Activated CD8+ T cell	0.19	0.21	0.17	1.61E-01
COAD	Activated dendritic cell	0.27	0.28	0.25	6.01E-02
COAD	$CD56^{bright}$ natural killer cell	0.26	0.25	0.25	3.90E-02
COAD	$CD56^{dim}$ natural killer cell	0.45	0.40	0.39	1.31E-04
COAD	Central memory CD4+ T cell	0.55	0.54	0.55	6.76E-01
COAD	Central memory CD8+ T cell	0.34	0.34	0.34	8.83E-01
COAD	Effector memory CD4+ T cell	0.06	0.10	0.12	4.12E-03
COAD	Effector memory CD8+ T cell	0.18	0.21	0.17	5.47E-01
COAD	Gamma delta T cell	0.29	0.28	0.28	2.15E-01
COAD	Immature dendritic cell	0.27	0.29	0.30	8.29E-02
COAD	MDSC	0.24	0.22	0.19	6.22E-02
COAD	Memory B cell	0.13	0.14	0.15	1.62E-04
COAD	Monocyte	0.41	0.40	0.39	4.50E-02
COAD	Natural killer cell	0.27	0.27	0.27	7.70E-01
COAD	Natural killer T cell	0.06	0.05	0.05	1.41E-01

© 2019 Xu X et al. *JAMA Network Open.*

COAD	Plasmacytoid dendritic cell	0.37	0.37	0.38	5.93E-01
COAD	Regulatory T cell	0.14	0.14	0.15	6.78E-01
COAD	T follicular helper cell	0.13	0.12	0.11	1.70E-01
COAD	Type 1 T helper cell	0.15	0.14	0.13	1.78E-01
COAD	Type 2 T helper cell	0.03	0.08	0.07	3.72E-02
HNSC	Activated CD4+ T cell	0.22	0.20	0.19	2.47E-03
HNSC	Activated CD8+ T cell	0.25	0.17	0.15	3.13E-19
HNSC	Activated dendritic cell	0.28	0.25	0.23	3.09E-15
HNSC	CD56^{bright} natural killer cell	0.31	0.30	0.29	5.75E-10
HNSC	CD56^{dim} natural killer cell	0.36	0.36	0.35	4.98E-01
HNSC	Central memory CD4+ T cell	0.59	0.58	0.56	4.85E-10
HNSC	Central memory CD8+ T cell	0.37	0.37	0.36	4.95E-02
HNSC	Effector memory CD4+ T cell	0.19	0.20	0.19	8.72E-01
HNSC	Effector memory CD8+ T cell	0.33	0.29	0.25	1.01E-17
HNSC	Gamma delta T cell	0.31	0.29	0.27	1.16E-09
HNSC	Immature dendritic cell	0.38	0.39	0.38	3.40E-01
HNSC	MDSC	0.37	0.29	0.24	2.11E-17
HNSC	Memory B cell	0.13	0.12	0.12	2.92E-01
HNSC	Monocyte	0.37	0.36	0.35	2.28E-09
HNSC	Natural killer cell	0.28	0.26	0.25	1.69E-05

© 2019 Xu X et al. *JAMA Network Open.*

HNSC	Natural killer T cell	0.13	0.11	0.09	9.38E-12
HNSC	Plasmacytoid dendritic cell	0.42	0.42	0.41	1.81E-01
HNSC	Regulatory T cell	0.23	0.18	0.13	5.87E-11
HNSC	T follicular helper cell	0.19	0.17	0.15	9.16E-08
HNSC	Type 1 T helper cell	0.18	0.16	0.13	1.55E-11
HNSC	Type 2 T helper cell	0.16	0.17	0.15	2.86E-01
KIRC	Activated CD8+ T cell	0.30	0.22	0.13	1.22E-17
KIRC	Activated dendritic cell	0.32	0.29	0.25	7.56E-20
KIRC	$CD56^{bright}$ natural killer cell	0.25	0.24	0.22	1.62E-16
KIRC	$CD56^{dim}$ natural killer cell	0.40	0.40	0.39	2.05E-01
KIRC	Central memory CD4+ T cell	0.60	0.59	0.56	4.58E-10
KIRC	Central memory CD8+ T cell	0.37	0.37	0.34	9.71E-03
KIRC	Effector memory CD4+ T cell	0.25	0.26	0.24	1.31E-01
KIRC	Effector memory CD8+ T cell	0.36	0.33	0.30	3.14E-10
KIRC	Gamma delta T cell	0.31	0.30	0.27	9.25E-10
KIRC	Immature dendritic cell	0.39	0.39	0.38	1.10E-02
KIRC	MDSC	0.43	0.37	0.28	3.02E-20
KIRC	Memory B cell	0.13	0.12	0.12	5.34E-03
KIRC	Monocyte	0.42	0.41	0.40	3.23E-06
KIRC	Natural killer cell	0.38	0.38	0.36	2.54E-04

© 2019 Xu X et al. *JAMA Network Open*.

KIRC	Natural killer T cell	0.16	0.15	0.13	3.33E-06
KIRC	Plasmacytoid dendritic cell	0.45	0.45	0.45	3.12E-01
KIRC	Regulatory T cell	0.28	0.26	0.22	1.44E-06
KIRC	T follicular helper cell	0.25	0.23	0.20	2.29E-13
KIRC	Type 1 T helper cell	0.24	0.22	0.19	1.41E-14
KIRC	Type 2 T helper cell	0.12	0.11	0.10	1.01E-01
LGG	Activated dendritic cell	0.21	0.16	0.15	6.58E-28
LGG	$CD56^{bright}$ natural killer cell	0.24	0.26	0.24	1.86E-03
LGG	$CD56^{dim}$ natural killer cell	0.34	0.31	0.32	9.05E-08
LGG	Central memory CD4++ T cell	0.48	0.44	0.43	1.29E-22
LGG	Central memory CD8+ T cell	0.24	0.21	0.20	1.52E-12
LGG	Effector memory CD4+ T cell	0.26	0.24	0.27	5.80E-03
LGG	Gamma delta T cell	0.16	0.13	0.12	6.87E-15
LGG	Immature dendritic cell	0.35	0.32	0.33	2.07E-24
LGG	Monocyte	0.43	0.43	0.43	3.39E-02
LGG	Natural killer cell	0.28	0.22	0.22	1.13E-31
LGG	Plasmacytoid dendritic cell	0.41	0.38	0.38	5.44E-30
LGG	T follicular helper cell	0.23	0.19	0.19	3.70E-19
LGG	Type 1 T helper cell	0.12	0.06	0.07	1.61E-42
LGG	Type 2 T helper cell	0.12	0.11	0.11	2.75E-04

© 2019 Xu X et al. *JAMA Network Open.*

LUAD	Activated CD4+ T cell	0.14	0.19	0.16	8.58E-04
LUAD	Activated CD8+ T cell	0.20	0.22	0.16	9.64E-01
LUAD	Activated dendritic cell	0.30	0.30	0.25	3.16E-05
LUAD	$CD56^{bright}$ natural killer cell	0.25	0.25	0.23	1.64E-02
LUAD	$CD56^{dim}$ natural killer cell	0.38	0.39	0.38	2.21E-01
LUAD	Central memory CD4+ T cell	0.60	0.60	0.57	1.50E-04
LUAD	Central memory CD8+ T cell	0.38	0.37	0.33	7.79E-04
LUAD	Effector memory CD4+ T cell	0.18	0.17	0.16	6.38E-03
LUAD	Effector memory CD8+ T cell	0.34	0.33	0.24	1.67E-06
LUAD	Gamma delta T cell	0.30	0.29	0.26	8.24E-03
LUAD	Immature dendritic cell	0.42	0.40	0.38	4.80E-09
LUAD	MDSC	0.40	0.39	0.28	3.57E-05
LUAD	Monocyte	0.41	0.40	0.38	1.20E-05
LUAD	Natural killer cell	0.31	0.31	0.27	9.23E-05
LUAD	Natural killer T cell	0.10	0.11	0.07	1.13E-01
LUAD	Plasmacytoid dendritic cell	0.42	0.41	0.38	2.99E-05
LUAD	Regulatory T cell	0.30	0.28	0.19	5.39E-05
LUAD	T follicular helper cell	0.22	0.20	0.16	5.67E-11
LUAD	Type 1 T helper cell	0.19	0.19	0.14	2.03E-03
LUAD	Type 2 T helper cell	0.15	0.15	0.15	5.81E-01

© 2019 Xu X et al. *JAMA Network Open.*

LUSC	Activated CD4+ T cell	0.25	0.20	0.21	3.37E-12
LUSC	Activated CD8+ T cell	0.22	0.14	0.17	1.88E-18
LUSC	Activated dendritic cell	0.28	0.22	0.23	3.80E-45
LUSC	$CD56^{bright}$ natural killer cell	0.27	0.27	0.28	5.93E-02
LUSC	$CD56^{dim}$ natural killer cell	0.35	0.32	0.34	2.50E-09
LUSC	Central memory CD4+ T cell	0.56	0.52	0.52	1.92E-30
LUSC	Central memory CD8+ T cell	0.37	0.33	0.34	4.43E-16
LUSC	Effector memory CD4+ T cell	0.19	0.17	0.17	5.81E-11
LUSC	Effector memory CD8+ T cell	0.32	0.21	0.22	9.75E-44
LUSC	Gamma delta T cell	0.29	0.24	0.25	3.61E-28
LUSC	Immature dendritic cell	0.38	0.36	0.36	2.00E-11
LUSC	MDSC	0.39	0.24	0.25	4.27E-40
LUSC	Memory B cell	0.11	0.11	0.11	4.94E-01
LUSC	Monocyte	0.36	0.33	0.33	1.46E-19
LUSC	Natural killer cell	0.28	0.23	0.23	6.95E-33
LUSC	Natural killer T cell	0.13	0.08	0.09	2.89E-26
LUSC	Plasmacytoid dendritic cell	0.40	0.37	0.38	5.80E-19
LUSC	Regulatory T cell	0.29	0.15	0.17	1.62E-38
LUSC	T follicular helper cell	0.19	0.13	0.14	4.45E-36
LUSC	Type 1 T helper cell	0.19	0.12	0.13	1.63E-34

© 2019 Xu X et al. *JAMA Network Open.*

LUSC	Type 2 T helper cell	0.16	0.13	0.14	4.46E-11
PRAD	Activated CD8+ T cell	0.16	0.17	0.14	7.22E-01
PRAD	Activated dendritic cell	0.19	0.22	0.20	2.22E-06
PRAD	$CD56^{bright}$ natural killer cell	0.30	0.31	0.30	2.17E-02
PRAD	$CD56^{dim}$ natural killer cell	0.30	0.31	0.30	2.62E-03
PRAD	Central memory CD4+ T cell	0.50	0.53	0.51	4.28E-07
PRAD	Central memory CD8+ T cell	0.23	0.24	0.24	2.55E-06
PRAD	Effector memory CD4+ T cell	0.14	0.16	0.16	5.23E-08
PRAD	Effector memory CD8+ T cell	0.15	0.19	0.16	2.26E-06
PRAD	Gamma delta T cell	0.17	0.18	0.17	2.34E-01
PRAD	Immature dendritic cell	0.35	0.36	0.36	7.58E-07
PRAD	Memory B cell	0.05	0.05	0.06	1.09E-02
PRAD	Monocyte	0.43	0.42	0.42	2.02E-01
PRAD	Natural killer cell	0.25	0.28	0.27	1.22E-14
PRAD	Plasmacytoid dendritic cell	0.40	0.41	0.41	4.11E-07
PRAD	T follicular helper cell	0.11	0.14	0.13	4.61E-10
PRAD	Type 1 T helper cell	0.06	0.09	0.07	2.79E-08
SKCM	Activated CD4+ T cell	0.24	0.21	0.14	2.44E-13
SKCM	Activated CD8+ T cell	0.28	0.16	0.12	2.51E-24

© 2019 Xu X et al. *JAMA Network Open.*

SKCM	Activated dendritic cell	0.28	0.23	0.19	1.45E-32
SKCM	CD56^{bright} natural killer cell	0.25	0.23	0.24	2.32E-09
SKCM	CD56^{dim} natural killer cell	0.34	0.34	0.33	2.05E-02
SKCM	Central memory CD4+ T cell	0.54	0.51	0.48	1.63E-22
SKCM	Central memory CD8+ T cell	0.34	0.33	0.30	1.33E-09
SKCM	Effector memory CD4+ T cell	0.17	0.17	0.13	1.30E-05
SKCM	Effector memory CD8+ T cell	0.34	0.24	0.19	4.02E-33
SKCM	Gamma delta T cell	0.29	0.28	0.26	5.88E-09
SKCM	Immature dendritic cell	0.36	0.35	0.32	5.15E-12
SKCM	MDSC	0.38	0.26	0.19	9.97E-26
SKCM	Memory B cell	0.18	0.19	0.17	3.63E-01
SKCM	Monocyte	0.42	0.40	0.39	1.96E-15
SKCM	Natural killer cell	0.32	0.28	0.25	2.93E-24
SKCM	Natural killer T cell	0.13	0.10	0.07	1.57E-18
SKCM	Plasmacytoid dendritic cell	0.42	0.41	0.40	4.31E-09
SKCM	Regulatory T cell	0.27	0.20	0.14	5.36E-19
SKCM	T follicular helper cell	0.23	0.18	0.14	1.79E-24
SKCM	Type 1 T helper cell	0.19	0.15	0.11	1.49E-22
SKCM	Type 2 T helper cell	0.18	0.19	0.17	6.74E-01
STAD	Activated CD4+ T cell	0.24	0.23	0.28	2.27E-08

© 2019 Xu X et al. *JAMA Network Open.*

STAD	Activated CD8+ T cell	0.08	0.16	0.27	2.95E-24
STAD	Activated dendritic cell	0.19	0.25	0.31	1.76E-28
STAD	CD56^{bright} natural killer cell	0.18	0.18	0.26	8.35E-06
STAD	CD56^{dim} natural killer cell	0.39	0.38	0.45	1.88E-01
STAD	Central memory CD4+ T cell	0.51	0.55	0.57	3.60E-24
STAD	Central memory CD8+ T cell	0.29	0.33	0.42	6.85E-16
STAD	Effector memory CD4+ T cell	0.16	0.21	0.13	7.31E-10
STAD	Effector memory CD8+ T cell	0.13	0.25	0.30	2.27E-41
STAD	Gamma delta T cell	0.19	0.21	0.26	2.05E-08
STAD	Immature dendritic cell	0.31	0.27	0.26	2.31E-03
STAD	MDSC	0.08	0.20	0.39	1.54E-33
STAD	Memory B cell	0.15	0.12	0.08	7.92E-04
STAD	Monocyte	0.32	0.40	0.42	1.02E-17
STAD	Natural killer cell	0.29	0.31	0.34	8.21E-27
STAD	Natural killer T cell	0.09	0.10	0.09	8.02E-23
STAD	Plasmacytoid dendritic cell	0.39	0.42	0.38	7.05E-07
STAD	Regulatory T cell	0.02	0.10	0.19	1.05E-21
STAD	T follicular helper cell	0.07	0.15	0.20	4.73E-24
STAD	Type 1 T helper cell	0.09	0.18	0.19	6.06E-32
STAD	Type 2 T helper cell	0.18	0.14	0.09	1.20E-07

© 2019 Xu X et al. *JAMA Network Open.*

THCA	Activated CD8+ T cell	0.19	0.09	0.06	8.27E-34
THCA	Activated dendritic cell	0.26	0.21	0.17	4.08E-45
THCA	CD56^{bright} natural killer cell	0.26	0.23	0.22	2.18E-51
THCA	CD56^{dim} natural killer cell	0.38	0.37	0.32	3.02E-26
THCA	Central memory CD4+ T cell	0.58	0.55	0.50	1.07E-45
THCA	Central memory CD8+ T cell	0.36	0.32	0.30	6.76E-28
THCA	Effector memory CD4+ T cell	0.19	0.18	0.17	1.59E-09
THCA	Effector memory CD8+ T cell	0.29	0.22	0.12	1.42E-44
THCA	Gamma delta T cell	0.25	0.22	0.18	1.72E-33
THCA	Immature dendritic cell	0.41	0.39	0.34	1.85E-28
THCA	Monocyte	0.44	0.42	0.42	7.49E-24
THCA	Natural killer cell	0.34	0.33	0.28	2.07E-20
THCA	Plasmacytoid dendritic cell	0.41	0.40	0.39	1.80E-21
THCA	T follicular helper cell	0.24	0.21	0.17	5.73E-30
THCA	Type 1 T helper cell	0.18	0.14	0.11	4.66E-33
THCA	Type 2 T helper cell	0.07	0.06	0.04	2.26E-05
UCEC	Activated CD8+ T cell	0.20	0.17	0.14	2.71E-02
UCEC	Activated dendritic cell	0.25	0.22	0.19	1.66E-05

© 2019 Xu X et al. *JAMA Network Open*.

UCEC	CD56^{bright} natural killer cell	0.25	0.25	0.23	3.46E-04
UCEC	CD56^{dim} natural killer cell	0.38	0.33	0.33	1.61E-07
UCEC	Central memory CD4+ T cell	0.49	0.49	0.47	6.55E-02
UCEC	Central memory CD8+ T cell	0.24	0.23	0.22	1.54E-02
UCEC	Effector memory CD4+ T cell	0.05	0.09	0.08	1.34E-03
UCEC	Effector memory CD8+ T cell	0.21	0.19	0.14	2.98E-03
UCEC	Gamma delta T cell	0.21	0.22	0.21	7.41E-01
UCEC	Immature dendritic cell	0.30	0.32	0.30	2.00E-01
UCEC	MDSC	0.28	0.20	0.15	5.91E-05
UCEC	Memory B cell	0.08	0.06	0.07	1.42E-01
UCEC	Monocyte	0.36	0.35	0.34	1.19E-03
UCEC	Natural killer cell	0.26	0.24	0.23	5.15E-04
UCEC	Plasmacytoid dendritic cell	0.35	0.36	0.35	4.81E-01
UCEC	T follicular helper cell	0.14	0.10	0.09	2.29E-05
UCEC	Type 1 T helper cell	0.12	0.10	0.08	5.12E-04
UCEC	Type 2 T helper cell	0.08	0.09	0.09	2.29E-02

Note: p-values represent the significant differences for each type of immune cells between the TIME-rich and TIME-intermediate/-poor subtypes.

© 2019 Xu X et al. *JAMA Network Open.*

eTable 4. Fractions of the Patients in TIME-Rich, TIME-Intermediate, and TIME-Poor Subtype in Cancers

Cancer	Patient (n)	TIME-rich (%)	TIME-intermediate (%)	TIME-poor (%)
UCEC	365	7.5	20.3	72.3
LUAD	512	17.4	52.5	30.1
BLCA	408	19.4	51.5	29.2
BRCA	314	22.0	37.2	40.8
STAD	374	22.2	11.5	66.3
KIRC	530	23.8	38.7	37.5
PRAD	497	26.4	13.1	60.6
COAD	87	26.4	16.1	57.5
HNSC	412	28.6	39.3	32.0
THCA	498	29.3	44.8	25.9
LUSC	501	30.7	40.1	29.1
SKCM	364	34.3	29.1	36.5
LGG	511	41.7	34.1	24.3

© 2019 Xu X et al. *JAMA Network Open.*

eTable 5. NK Defective Genes in Each Cancer Type

Cancer type	Gene	Annotation
LGG	CMA1	NK cell
	CSF2	NK cell+NKT cell+γδ T cell
	FASLG	NK cell+NKT cell
	IKZF3	NK cell+NKT cell
	KIR2DL4	NK cell+NKT cell
	KIR3DL3	NK cell+NKT cell
	KLHL30	NK cell
	NCR1	NK cell+γδ T cell
	NCR2	NK cell
	NCR3	NK cell
	SH2D1B	NK cell+γδ T cell
BRCA	C17orf66	NK cell+NKT cell
	CD244	NK cell+NKT cell+γδ T cell
	CSF2	NK cell+NKT cell+γδ T cell
	KHDC1	NK cell
	KIR2DL3	NK cell+NKT cell
	KIR2DL4	NK cell+NKT cell
	KIR3DL2	NK cell+NKT cell
	KIR3DL3	NK cell+NKT cell
	KLHL30	NK cell
	KLRF1	NK cell
	NCR3	NK cell
	SH2D1B	NK cell+γδ T cell
HNSC	C17orf66	NK cell+NKT cell
	CD244	NK cell+NKT cell+γδ T cell
	CDC20B	NK cell+γδ T cell

© 2019 Xu X et al. *JAMA Network Open.*

	CHRNE	NK cell
	CLNK	NK cell+γδ T cell
	CMA1	NK cell
	KIR2DL3	NK cell+NKT cell
	KIR2DL4	NK cell+NKT cell
	KIR3DL1	NK cell+NKT cell
	KIR3DL3	NK cell+NKT cell
	KLRF1	NK cell
	NCR2	NK cell
	NCR3	NK cell
BLCA	C17orf66	NK cell+NKT cell
	CDC20B	NK cell+γδ T cell
	CLNK	NK cell+γδ T cell
	CMA1	NK cell
	CSF2	NK cell+NKT cell+γδ T cell
	IKZF3	NK cell+NKT cell
	KHDC1	NK cell
	KIR2DL1	NK cell+NKT cell
	KIR2DL3	NK cell+NKT cell
	KIR3DL1	NK cell+NKT cell
	KIR3DL2	NK cell+NKT cell
	KIR3DL3	NK cell+NKT cell
	KLHL30	NK cell
	KLRB1	NK cell+NKT cell+γδ T cell
	NCR2	NK cell
	NCR3	NK cell
	SH2D1B	NK cell+γδ T cell
LUSC	C17orf66	NK cell+NKT cell
	CD244	NK cell+NKT cell+γδ T cell

© 2019 Xu X et al. *JAMA Network Open.*

	CDC20B	NK cell+γδ T cell
	CHRNE	NK cell
	CLNK	NK cell+γδ T cell
	CMA1	NK cell
	CSF2	NK cell+NKT cell+γδ T cell
	FASLG	NK cell+NKT cell
	KIR2DL1	NK cell+NKT cell
	KIR2DL3	NK cell+NKT cell
	KIR3DL1	NK cell+NKT cell
	KIR3DL2	NK cell+NKT cell
	KLHL30	NK cell
	KLRF1	NK cell
	NCR2	NK cell
	SH2D1B	NK cell+γδ T cell
LUAD	C17orf66	NK cell+NKT cell
	CD244	NK cell+NKT cell+γδ T cell
	CDC20B	NK cell+γδ T cell
	CMA1	NK cell
	CSF2	NK cell+NKT cell+γδ T cell
	FASLG	NK cell+NKT cell
	KHDC1	NK cell
	KIR2DL1	NK cell+NKT cell
	KIR2DL4	NK cell+NKT cell
	KIR3DL2	NK cell+NKT cell
	KIR3DL3	NK cell+NKT cell
	KLHL30	NK cell
	KLRF1	NK cell
	NCR1	NK cell+γδ T cell
	NCR2	NK cell

© 2019 Xu X et al. *JAMA Network Open.*

	NCR3	NK cell
	SH2D1B	NK cell+γδ T cell
PRAD	CD244	NK cell+NKT cell+γδ T cell
	CLNK	NK cell+γδ T cell
	CMA1	NK cell
	CSF2	NK cell+NKT cell+γδ T cell
	IKZF3	NK cell+NKT cell
	KIR2DL1	NK cell+NKT cell
	KIR2DL3	NK cell+NKT cell
	KIR2DL4	NK cell+NKT cell
	KIR3DL1	NK cell+NKT cell
	KIR3DL3	NK cell+NKT cell
	KLHL30	NK cell
	KLRF1	NK cell
	NCR2	NK cell
	NCR3	NK cell
	SH2D1B	NK cell+γδ T cell
STAD	KLRF1	NK cell
	NCR2	NK cell
	NCR3	NK cell
	C17orf66	NK cell+NKT cell
	KIR2DL1	NK cell+NKT cell
	KIR3DL1	NK cell+NKT cell
	KIR3DL3	NK cell+NKT cell
	CD244	NK cell+NKT cell+γδ T cell
	CDC20B	NK cell+γδ T cell
	CLNK	NK cell+γδ T cell
	SH2D1B	NK cell+γδ T cell
UCEC	CMA1	NK cell

© 2019 Xu X et al. *JAMA Network Open.*

	KHDC1	NK cell
	KLRF1	NK cell
	NCR2	NK cell
	NCR3	NK cell
	C17orf66	NK cell+NKT cell
	FASLG	NK cell+NKT cell
	KIR2DL1	NK cell+NKT cell
	KIR2DL3	NK cell+NKT cell
	KIR2DL4	NK cell+NKT cell
	KIR3DL1	NK cell+NKT cell
	KIR3DL2	NK cell+NKT cell
	CD244	NK cell+NKT cell+γδ T cell
	CSF2	NK cell+NKT cell+γδ T cell
	CLNK	NK cell+γδ T cell
	SH2D1B	NK cell+γδ T cell
	ANGPT1	NK cell+NKT cell
SKCM	C17orf66	NK cell+NKT cell
	CLNK	NK cell+γδ T cell
	CMA1	NK cell
	CSF2	NK cell+NKT cell+γδ T cell
	KIR3DL2	NK cell+NKT cell
	KLHL30	NK cell
	KLRF1	NK cell
	NCR2	NK cell
KIRC	C17orf66	NK cell+NKT cell
	CDC20B	NK cell+γδ T cell
	CLNK	NK cell+γδ T cell
	CMA1	NK cell
	CSF2	NK cell+NKT cell+γδ T cell

© 2019 Xu X et al. *JAMA Network Open.*

	KHDC1	NK cell
	KIR2DL1	NK cell+NKT cell
	KIR2DL3	NK cell+NKT cell
	KIR2DL4	NK cell+NKT cell
	KIR3DL2	NK cell+NKT cell
	KIR3DL3	NK cell+NKT cell
	KLHL30	NK cell
	KLRF1	NK cell
	NCR2	NK cell
	STYK1	NK cell+γδ T cell
THCA	CHRNE	NK cell
	CLNK	NK cell+γδ T cell
	CMA1	NK cell
	CSF2	NK cell+NKT cell+γδ T cell
	FASLG	NK cell+NKT cell
	KIR2DL1	NK cell+NKT cell
	KIR2DL3	NK cell+NKT cell
	KIR3DL3	NK cell+NKT cell
	STYK1	NK cell+γδ T cell

© 2019 Xu X et al. *JAMA Network Open.*

eTable 6. Experimental Evidence of the NK Cell Defective Genes for Tumor Surveillance

Gene	Tumor immune surveillance	Reference
CD244	CD244 stimulates NK cell activation to overcome resistance of leukemia and neuroblastoma cells	[22]
NCR1	Tumors in the absence of NCR1 grow faster in mice	[23]

© 2019 Xu X et al. *JAMA Network Open.*

eTable 7. ITAM-Signaling Genes Associated With Patients' Survival and Abundance of TILs in Cancers

Cancer type	Gene
LGG	ULBP2
	CD226
BRCA	ULBP3
	ITGB2
HNSC	PLCG2
	ITGB2
BLCA	ULBP2
LUSC	CD226
	HLA-E
LUAD	ICAM1
PRAD	CD226
	ICAM1
	ITGB2
	VAV1
STAD	PLCG1
	ULBP2
	ZAP70
UCEC	CD226
	PLCG2
	VAV1
SKCM	CD226
	ULBP1
	ULBP3
KIRC	HLA-E
THCA	PLCG1
	ULBP1

© 2019 Xu X et al. *JAMA Network Open.*

	ITGB2

© 2019 Xu X et al. *JAMA Network Open.*

eTable 8. Abundance of TILs in Tumors Stratified by the Expression of NK Cell Ligand Genes of Tumors for the Bottom 10% and Top 10% of Patients Ranked by the Mumber of NK-Defective Genes

Cancer	Cell type	p-value (bottom 10%)	Ratio*	p-value (top 10%)	Ratio*
BLCA	Activated CD8 T	5.9E-03	2.41	NS	-
	γδ T cell	4.9E-06	2.88	NS	-
	NK cell	6.6E-09	2.65	6.7E-03	1.26
	NKT cell	9.8E-12	1.27	1.0E-05	1.86
BRCA	Activated CD8 T	4.2E-02	1.16	NS	-
	γδ T cell	1.4E-02	1.43	NS	-
	NK cell	1.1E-04	1.35	NS	-
	NKT cell	2.1E-04	1.32	NS	-
HNSC	Activated CD8 T	8.7E-03	2.31	NS	-
	γδ T cell	1.9E-02	2.73	NS	-
	NK cell	1.4E-05	2.54	NS	-
	NKT cell	5.7E-04	2.01	NS	-
KIRC	Activated CD8 T	NS	-	NS	-
	γδ T cell	3.8E-03	4.19	NS	-
	NK cell	9.6E-03	4.67	NS	-
	NKT cell	8.1E-03	19.98	NS	-
LGG	Activated CD8 T	4.2E-08	1.57	NS	-
	γδ T cell	1.4E-10	1.42	2.6E-02	1.36
	NK cell	3.5E-10	1.73	NS	-
	NKT cell	1.3E-15	2.81	NS	-
LUAD	Activated CD8 T	NS	-	NS	-
	γδ T cell	3.2E-02	2.44	1.2E-02	1.05
	NK cell	1.1E-03	2.34	2.7E-03	1.09

© 2019 Xu X et al. *JAMA Network Open.*

	NKT cell	NS	-	5.4E-05	1.88
LUSC	Activated CD8 T	2.4E-03	1.22	1.0E-03	3.03
	γδ T cell	2.0E-04	1.62	1.2E-02	4.76
	NK cell	1.2E-08	1.49	1.8E-04	4.35
	NKT cell	2.1E-09	1.08	2.2E-04	3.33
PRAD	Activated CD8 T	NS	-	3.8E-02	1.17
	γδ T cell	2.4E-02	4.42	1.7E-04	1.33
	NK cell	1.8E-03	4.21	4.0E-05	1.35
	NKT cell	NS	-	NS	-
SKCM	Activated CD8 T	NS	-	NS	-
	γδ T cell	NS	-	NS	-
	NK cell	NS	-	NS	-
	NKT cell	3.2E-02	3.25	NS	-
STAD	Activated CD8 T	1.8E-02	5.35	2.4E-02	2.51
	γδ T cell	5.0E-01	6.69	NS	-
	NK cell	8.0E-03	6.15	1.1E-02	1.45
	NKT cell	5.0E-03	4.49	1.3E-02	1.23
THCA	Activated CD8 T	NS	-	2.5E-02	1.22
	γδ T cell	NS	-	NS	-
	NK cell	2.5E-02	2.32	NS	-
	NKT cell	NS	-	NS	-
UCEC	Activated CD8 T	NS	-	NS	-
	γδ T cell	NS	-	NS	-
	NK cell	NS	-	NS	-
	NKT cell	3.2E-02	1.7-E02	NS	-

Note: ratio is the TIL's abundance of the high-expression group/the low-expression group.

© 2019 Xu X et al. *JAMA Network Open.*

eTable 9. Clustering Analysis for the Melanoma (SKCM) and Gastric Cancer (STAD) Samples in Immune-Checkpoint Therapy (ICT) Trials

Cancer type	Group	Number of responding patients	Number of non-responding	Enrichment ratio*
SKCM	TIME-rich	7	22	1.24
	TIME-intermediate/-poor	3	17	0.69
STAD	TIME-rich	7	11	1.86
	TIME-intermediate/-poor	5	24	0.61

*Ratio of the enrichment of responding patients in each group to that in all patient

© 2019 Xu X et al. *JAMA Network Open.*

eReferences

1. Bezman NA, Kim CC, Sun JC, et al. Molecular definition of the identity and activation of natural killer cells. *Nature immunology.* 2012;13(10):1000-1009.
2. Heng TS, Painter MW, Immunological Genome Project C. The Immunological Genome Project: networks of gene expression in immune cells. *Nature immunology.* 2008;9(10):1091-1094.
3. Koboldt DC, Zhang Q, Larson DE, et al. VarScan 2: somatic mutation and copy number alteration discovery in cancer by exome sequencing. *Genome research.* 2012;22(3):568-576.
4. Kircher M, Witten DM, Jain P, O'Roak BJ, Cooper GM, Shendure J. A general framework for estimating the relative pathogenicity of human genetic variants. *Nature genetics.* 2014;46(3):310-315.
5. Li H, Durbin R. Fast and accurate short read alignment with Burrows-Wheeler transform. *Bioinformatics.* 2009;25(14):1754-1760.
6. Li H. A statistical framework for SNP calling, mutation discovery, association mapping and population genetical parameter estimation from sequencing data. *Bioinformatics.* 2011;27(21):2987-2993.
7. Rock KL, Reits E, Neefjes J. Present Yourself! By MHC Class I and MHC Class II Molecules. *Trends in immunology.* 2016;37(11):724-737.
8. Charoentong P, Finotello F, Angelova M, et al. Pan-cancer Immunogenomic Analyses Reveal Genotype-Immunophenotype Relationships and Predictors of Response to Checkpoint Blockade. *Cell reports.* 2017;18(1):248-262.
9. Patel SJ, Sanjana NE, Kishton RJ, et al. Identification of essential genes for cancer immunotherapy. *Nature.* 2017;548(7669):537-+.
10. Pan D, Kobayashi A, Jiang P, et al. A major chromatin regulator determines resistance of tumor cells to T cell-mediated killing. *Science.* 2018;359(6377):770-+.
11. Brock G, Datta S, Pihur V, Datta S. clValid: An R package for cluster validation. *J Stat Softw.* 2008;25(4):1-22.
12. Zou J, Wang E. eTumorRisk, an algorithm predicts cancer risk based on co-mutated gene networks in an individual's germline genome. *bioRxiv.* 2018:393090.
13. Ohkuma T, Peters SAE, Woodward M. Sex differences in the association between diabetes and cancer: a systematic review and meta-analysis of 121 cohorts including 20 million individuals and one million events. *Diabetologia.* 2018;61(10):2140-2154.
14. Gross AL, Gallo JJ, Eaton WW. Depression and cancer risk: 24 years of follow-up of the Baltimore Epidemiologic Catchment Area sample. *Cancer causes & control : CCC.* 2010;21(2):191-199.
15. Batty GD, Russ TC, Stamatakis E, Kivimaki M. Psychological distress in relation to site specific cancer mortality: pooling of unpublished data from 16 prospective cohort studies. *Bmj.* 2017;356:j108.
16. Bahr I, Jahn J, Zipprich A, Pahlow I, Spielmann J, Kielstein H. Impaired natural killer cell subset phenotypes in human obesity. *Immunologic research.* 2018;66(2):234-244.
17. O'Shea D, Cawood TJ, O'Farrelly C, Lynch L. Natural killer cells in obesity: impaired function and increased susceptibility to the effects of cigarette smoke. *PloS one.* 2010;5(1):e8660.
18. Wyman PA, Moynihan J, Eberly S, et al. Association of family stress with natural killer cell activity and the frequency of illnesses in children. *Archives of pediatrics & adolescent medicine.* 2007;161(3):228-234.

© 2019 Xu X et al. *JAMA Network Open.*

19. Spranger S, Bao R, Gajewski TF. Melanoma-intrinsic beta-catenin signalling prevents anti-tumour immunity. *Nature.* 2015;523(7559):231-235.
20. Grasso CS, Giannakis M, Wells DK, et al. Genetic Mechanisms of Immune Evasion in Colorectal Cancer. *Cancer discovery.* 2018;8(6):730-749.
21. Bjorkstrom NK, Ljunggren HG, Michaelsson J. Emerging insights into natural killer cells in human peripheral tissues. *Nature reviews Immunology.* 2016;16(5):310-320.
22. Altvater B, Landmeier S, Pscherer S, et al. 2B4 (CD244) signaling by recombinant antigen-specific chimeric receptors costimulates natural killer cell activation to leukemia and neuroblastoma cells. *Clin Cancer Res.* 2009;15(15):4857-4866.
23. Elboim M, Gazit R, Gur C, Ghadially H, Betser-Cohen G, Mandelboim O. Tumor immunoediting by NKp46. *J Immunol.* 2010;184(10):5637-5644.

© 2019 Xu X et al. *JAMA Network Open.*
